# Long-term outcomes of passive immunotherapy for COVID-19: a pooled analysis of a large multinational platform randomized clinical trial

**DOI:** 10.1016/j.cmi.2025.02.002

**Published:** 2025-02-06

**Authors:** Ahmad Mourad, Gregory A. Grandits, Lianne K. Siegel, Nicole Engen, Christina Barkauskas, Nnakelu Eriobu, Mamta K. Jain, Tomas O. Jensen, Adit Ginde, Elizabeth Higgs, Daniel B. Knox, Jonathan Kitonsa, Kami Kim, Jakob J. Malin, Vasiliki Rapti, D. Ashley Price, Alfredo J. Mena Lora, Gail Mathews, Eleftherios Mylonakis, Thomas A. Murray, Uriel Sandkovsky, Roger Paredes, Srikanth Ramachandruni, Cavan Reilly, David Vock, John C. Williamson, Barnaby Edward Young, Wesley H. Self, Jens Lundgren, Thomas L. Holland

**Affiliations:** 1 Department of Medicine, Division of Infectious Diseases, Duke University School of Medicine, Durham, NC, USA; 2 Duke Clinical Research Institute, Durham NC, USA; 3 Division of Biostatistics and Health Data Science, School of Public Health, University of Minnesota, Minnesota, MN, USA; 4 Department of Medicine, Division of Pulmonary, Allergy, and Critical Care, Duke University School of Medicine, Durham, NC, USA; 5 Institute of Human Virology Nigeria, Abuja, Nigeria; 6 Department of Internal Medicine, Division of Infectious Diseases, University of Texas Southwestern Medical Center, Dallas, TX, USA; 7 Parkland Health, Dallas, TX, USA; 8 Centre of Excellence for Health, Immunity, and Infections, Rigshospitalet, University of Copenhagen, Copenhagen, Denmark; 9 Department of Emergency Medicine, University of Colorado School of Medicine, Aurora, CO, USA; 10 National Institute of Allergy and Infectious Diseases, National Institutes of Health, Bethesda, MD, USA; 11 Division of Pulmonary and Critical Care Medicine, Intermountain Medical Center, Murray, UT, USA; 12 Medical Research Council/Uganda Virus Research Institute, Entebbe, Uganda; 13 London School of Hygiene & Tropical Medicine Uganda Research Unit, Entebbe, Uganda; 14 Division of Infectious Disease and International Medicine, Department of Internal Medicine, University of South Florida and Tampa General Hospital, Tampa, FL, USA; 15 Department of Internal Medicine, Division of Infectious Diseases, Faculty of Medicine and University Hospital Cologne, University of Cologne, Cologne, Germany; 16 Third Department of Internal Medicine, School of Medicine, National & Kapodistrian University of Athens, Sotiria General Hospital, Athens, Greece; 17 Department of Infectious Diseases, Royal Victoria Infirmary, Newcastle upon Tyne Hospitals Foundation Trust, Newcastle upon Tyne, United Kingdom; 18 Division of Infectious Diseases, Department of Medicine, University of Illinois Chicago, Chicago, IL, USA; 19 Kirby Institute, University of New South Wales, Sydney, New South Wales, Australia; 20 Department of Medicine, Houston Methodist Hospital, Houston, TX, USA; 21 Division of Infectious Diseases, Department of Medicine, Baylor University Medical Center, Dallas, TX, USA; 22 Department of Infectious Diseases & IrsiCaixa, Hospital Universitari Germans Trias i Pujol, Badalona, Catalonia, Spain; 23 Center for Global Health and Diseases, Department of Pathology, Case Western Reserve University School of Medicine, Cleveland, OH, USA; 24 Christus Spohn Hospital, Corpus Christi, TX, USA; 25 Department of Internal Medicine, Section on Infectious Diseases, Wake Forest University School of Medicine, Winston-Salem, NC, USA; 26 National Centre for Infectious Diseases, Novena, Singapore; 27 Tan Tock Seng Hospital, Novena, Singapore; 28 Lee Kong Chian School of Medicine, Nanyang Technological University, Novena, Singapore; 29 Vanderbilt Institute for Clinical & Translational Research, Department of Emergency Medicine, Vanderbilt University Medical Center, Nashville, TN, USA; 30 Rigshospitalet, University of Copenhagen Department of Infectious Diseases, Centre of Excellence for Health, Immunity and Infections, Copenhagen, Denmark

**Keywords:** COVID-19, Monoclonal antibodies, Neutralizing antibodies, Passive immunotherapy, SARS-CoV-2

## Abstract

**Objectives:**

Passive immunotherapy, including monoclonal antibodies and neutralizing proteins, was used for the treatment of patients with COVID-19 during the pandemic. Accelerating COVID-19 Therapeutic Interventions and Vaccines–Therapeutics for Inpatients with COVID-19 (ACTIV-3/TICO) was a multinational, randomized placebo-controlled platform trial that evaluated the effectiveness of multiple passive immunotherapy agents in patients hospitalized with COVID-19. Given the long half-life of some agents studied, participants were followed for an extended period to assess the long-term efficacy and sustained safety of these agents.

**Methods:**

We conducted a pooled analysis of individual participant data from four trials of ACTIV-3/TICO: sotrovimab, amubarvimab–romlusevimab, tixagevimab–cilgavimab, and ensovibep. Cox proportional hazards models were conducted to compare time to mortality and time to mortality or rehospitalization between participants receiving active agents vs. placebo through 18 months.

**Results:**

A total of 2311 participants were enrolled between 16 December 2020 and 15 November 2021. Overall, 56.9% (1315/2311) received an active agent and 77.2% (1784/2311) of participants were unvaccinated for SARS-CoV-2. Median duration between symptom onset and enrolment was 8 days (interquartile range, 6–10), and most participants received remdesivir (92.1% [2129/2311]) and corticosteroids (70.4% [1627/2311]) before enrolment. There was no difference in mortality across all active (11.9% [157/1315]) vs. placebo (14.0% [139/996]) arms (hazard ratio, 0.87; 95% CI, 0.70–1.08). Furthermore, there was no difference in combined mortality or rehospitalization across all active (31.7% [417/1315]) vs. placebo (32.1% [320/996]) arms (hazard ratio, 0.96; 95% CI, 0.84–1.10).

**Discussion:**

In our large study of long half-life passive immunotherapy for hospitalized patients with COVID-19, we did not find evidence of a long-term effect on either mortality or rehospitalization.

**Trial registration:**

NCT04501978.

## Introduction

Several anti-SARS-CoV-2 monoclonal antibodies and novel neutralizing protein therapeutics (passive immunotherapy) have been studied for the treatment of patients with COVID-19 [1–10]. Early treatment with passive immunotherapy was effective at preventing progression to severe disease in outpatients with COVID-19 [6–10], and improved outcomes were seen among hospitalized patients seronegative for endogenous neutralizing antibodies to SARS-CoV-2 at the time of randomization [5,11]. With the emergence of new SARS-CoV-2 variants and increased population level seropositivity, whether from prior infection or vaccination, treatment with passive immunotherapy currently has a limited role in the management of patients with COVID-19 [12–14]. However, passive immunotherapy will likely again be an important treatment strategy in the early stages of future viral outbreaks. Understanding the long-term safety and efficacy of these therapeutics is therefore crucial for future pandemic response and preparedness. Importantly, the long-term outcomes of hospitalized patients with COVID-19 who received passive immunotherapy are yet to be explored.

The Accelerating COVID-19 Therapeutic Interventions and Vaccines–Therapeutics for Inpatients with COVID-19 (ACTIV-3/TICO) master protocol was a large, multinational, randomized placebo-controlled platform trial that evaluated the effectiveness of antiviral therapies, including passive immunotherapy, in patients hospitalized with COVID-19 [1–4,15]. The primary efficacy outcome of these ACTIV-3/TICO trials was time to sustained clinical recovery at 90 days from randomization, defined as the time between randomization and return to the patient’s residence before hospitalization (or an equivalent location that provided similar or less-intensive medical care) for 14 consecutive days. Secondary outcomes, including all-cause mortality, were followed 90 days after randomization.

Given the long half-lives (up to 90 days) of some of the agents (sotrovimab, amubarvimab–romlusevimab, tixagevimab–cilgavimab, and ensovibep) [16] studied in ACTIV-3/TICO, it was possible that short-term efficacy might be offset by long-term harms. Theoretical safety concerns for these agents included blunting of subsequent SARS-CoV-2 vaccine response and persistent end-organ effects. Expected findings that may corrob orate these concerns include increased mortality and rehospitalization over the longer follow-up period. Thus, long-term follow-up for these outcomes was increased to at least three times the half-life of agents studied in the ACTIV-3/TICO platform. Here, we report a pooled analysis of secondary long-term outcomes across four trials.

## Methods

### Ethical statement

The clinical trials were approved by central and/or local institutional review board at each study site. All patients provided written informed consent before enrolment.

### Clinical trial design and outcomes

The design, enrolment criteria, and the individual 90-day trial results for ACTIV-3/TICO have been previously published [1–4,15]. Briefly, the ACTIV-3/TICO master protocol was a large, multinational, platform, randomized placebo-controlled trial that evaluated the effectiveness of the following long half-life passive immunotherapeutic agents: sotrovimab (VIR-7831; Vir Biotechnology and GlaxoSmithKline), amubarvimab–romlusevimab (consisting of BRII-196 and BRII-198; Brii Biosciences), tixagevimab–cilgavimab (AZD7442 [Evusheld], consisting of AZD8895 and AZD1061; AstraZeneca), and ensovibep (MP0420; Molecular Partners). Participants were recruited from countries in Africa, Asia, Europe, and the United States (Table S1). Eligibility criteria were similar across the four trials [15] except that participants on non-invasive positive pressure ventilation or high-flow nasal cannula were only eligible for the tixagevimab–cilgavimab and ensovibep trials. Because several trials could enrol concurrently on the platform, patients who were eligible for multiple trials were first randomized to active agent or placebo (the odds of randomization to either arm varied over time depending on the number of active agents being evaluated), then to a specific agent/matched placebo. Participants who were randomized to receive a matched placebo served as shared controls for each trial for which they were eligible. Outcomes for the pooled analysis included all-cause mortality and a combined endpoint of mortality or rehospitalization through 18 months from randomization, and are reported here. Causes of death and primary cause for rehospitalizations (after day 90) were coded using the Medical Dictionary for Regulatory Activities version 23.1 (MedDRA), and summarized by System Organ Class (SOC). Hospitalizations during the first 90 days were not MedDRA coded.

### Laboratory measurements

Plasma samples were obtained from participants before randomization and analysed centrally for concentrations of neutralizing IgG antibodies against the receptor binding domain of the SARS-CoV-2 spike protein (GenScript SARS-CoV-2 Surrogate Virus Neutralization assay; GenScript, Piscataway, NJ, USA) and the SARS-CoV-2 nucleocapsid antigen (BioRad Platelia SARS-CoV-2 Total Ab assay; BioRad, Hercules, CA, USA).

### Statistical methods

Participants who were randomized into one of the four trials and received a full or partial infusion of either active agent or matched placebo were included in the pooled analysis (modified intention to treat). A Cox proportional hazards regression model, stratified by trial, was used to compare time with mortality, as well as a composite outcome of time to mortality or hospitalization, between participants receiving active agents vs. matched placebo through 18 months from enrolment. Participants who did not meet an outcome were censored at 18 months or the date of the last follow-up if they did not complete the final follow-up at 18 months. Hazard ratios are presented as active/placebo. To avoid counting participants more than once, participants randomized to placebo that were shared across trials were classified under the trial into which they were enrolled when pooling. These shared placebo participants were then weighted by the number of agents for which they served as controls to account for varying randomization ratios (active/placebo) over time. The treatment effect was compared between the four trials by adding a treatment-by-trial interaction term (3 DF) to the model (using agent-matched controls). Additionally, outcomes of participants receiving active agents or matched placebo were compared between two time periods: (1) up to 90 days from enrolment and (2) 91 days through 18 months from enrolment. An indicator of being in the latter time period was then treated as a time-dependent variable. A treatment by time period interaction term was added to assess differential treatment effects by time period.

A further subgroup analysis was conducted by participant serostatus at enrolment (i.e. baseline seropositive or seronegative for neutralizing IgG antibodies against the receptor binding domain of the SARS-CoV-2 spike protein). An interaction term between baseline serostatus and treatment arm was included to assess whether outcomes differed by baseline serostatus. In all models of the pooled trial data containing a treatment-covariate interaction term, the main effects of the terms included in the interaction were stratified by trial to avoid introducing aggregation bias because of the use of between-trial information [17,18].

Given that the tixagevimab–cilgavimab trial had the largest sample size, sensitivity analyses were performed excluding this trial from the pooled analysis. In addition to the pooled analyses, analyses were also conducted separately for each trial. In this case, the fully modified intention-to-treat cohort was used for each trial, with shared placebo participants contributing to multiple trials.

## Results

**Fig. 1.**
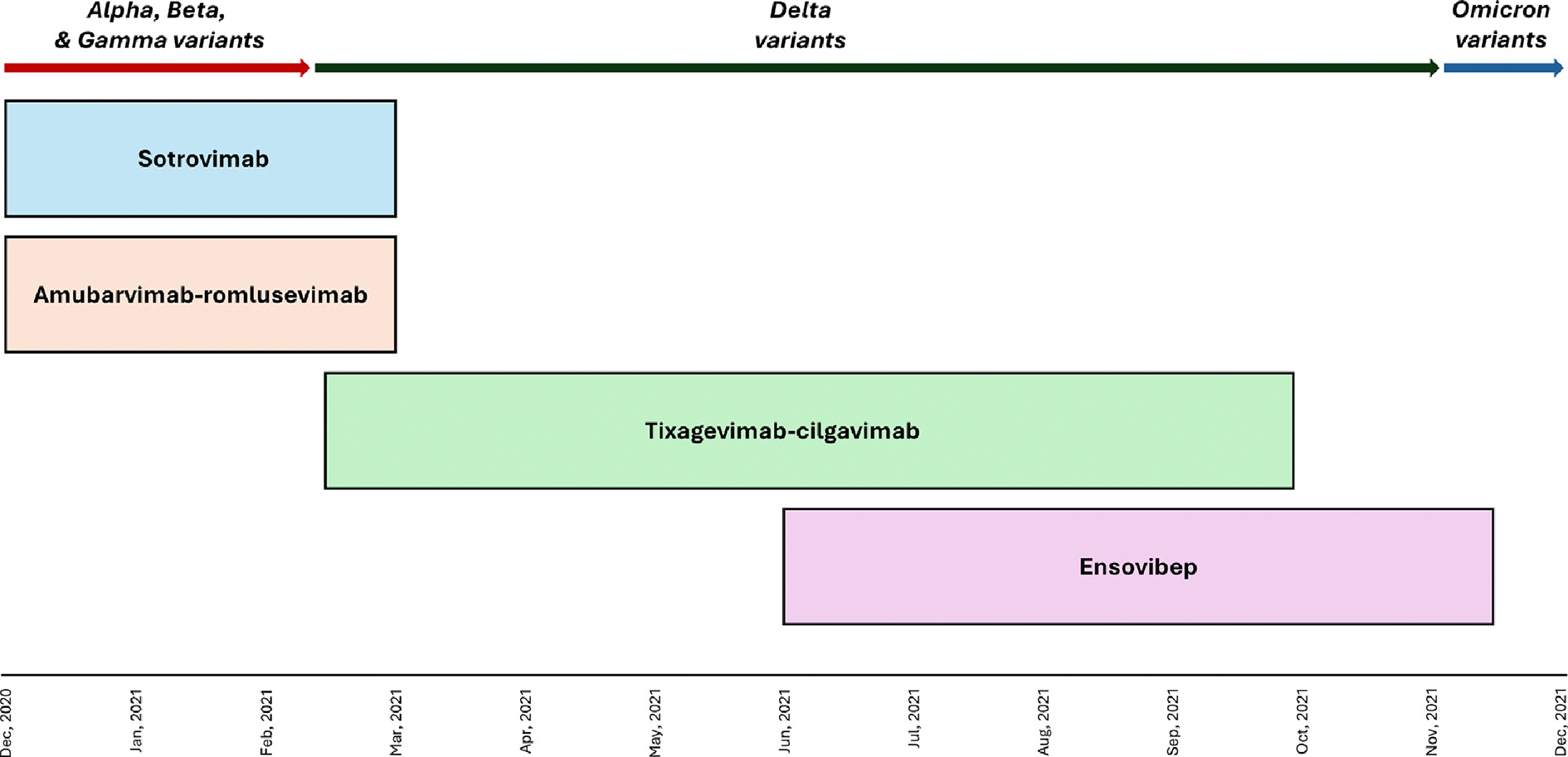
Enrolment periods for the four long half-life passive immunotherapy trials of ACTIV-3/TICO, and the predominant SARS-CoV-2 variants during those periods. ACTIV-3/TICO, Accelerating COVID-19 Therapeutic Interventions and Vaccines–Therapeutics for Inpatients with COVID-19.

Between 16 December 2020 and 15 November 2021, a total of 2311 participants were enrolled into one of four trials: 269 to sotrovimab (16 December 2020–1 March 2021), 262 to amubarvimab–romlusevimab (16 December 2020–1 March 2021), 1351 to tixagevimab–cilgavimab (10 February 2021–30 September 2021), and 429 to ensovibep (11 June 2021–15 November 2021) (Fig. 1). On the basis of prespecified criteria for futility, enrolment into the sotrovimab, amubarvimab–romlusevimab, and ensovibep trials was terminated early. Overall, 56.9% (1315/2311) of participants received an active agent, 57.9% were male (1337/2311), and the median age was 57 years (interquartile range, 46–68) (Table 1). The most common comorbidities were obesity (52.6% [1212/2311]), cardiovascular disease (46.9% [1085/2311]), and diabetes (28.1% [650/2311]). Approximately 9% (205/2311) of participants had an immune compromising condition or were receiving immune suppressive therapy, including anti-rejection medications. Median days since symptom onset was 8 (interquartile range, 6–10), and most participants were receiving remdesivir (92.1% [2129/2311]) and corticosteroids (70.4% [1627/2311]) before enrolment.

Although 77.2% (1784/2311) of participants were unvaccinated for SARS-CoV-2 at the time of enrolment across all trials, a greater proportion of participants were unvaccinated in the earlier trials (Table 1). Approximately half of the participants were seropositive for anti-spike neutralizing antibodies (51.8% [1157/2311]), and 62.8% (1403/2311) were positive for anti-nucleocapsid antibodies.

There were 296 deaths through 18 months of follow-up (12.8% [296/2311]). The most common causes of death by MedDRA SOC were respiratory, thoracic, mediastinal (42.6% [126/296]), infections and infestations (29.1% [86/296]), and cardiac (7.8% [23/296]) (Table S2). There were 608 total rehospitalizations between day 90 from enrolment through month 18. The most common causes for rehospitalization during this period were infections and infestations (20.2% [123/608]), respiratory, thoracic, mediastinal (13.7% [83/608]), and cardiac (11.2% [68/608]) (Table S3).

**Fig. 2.**
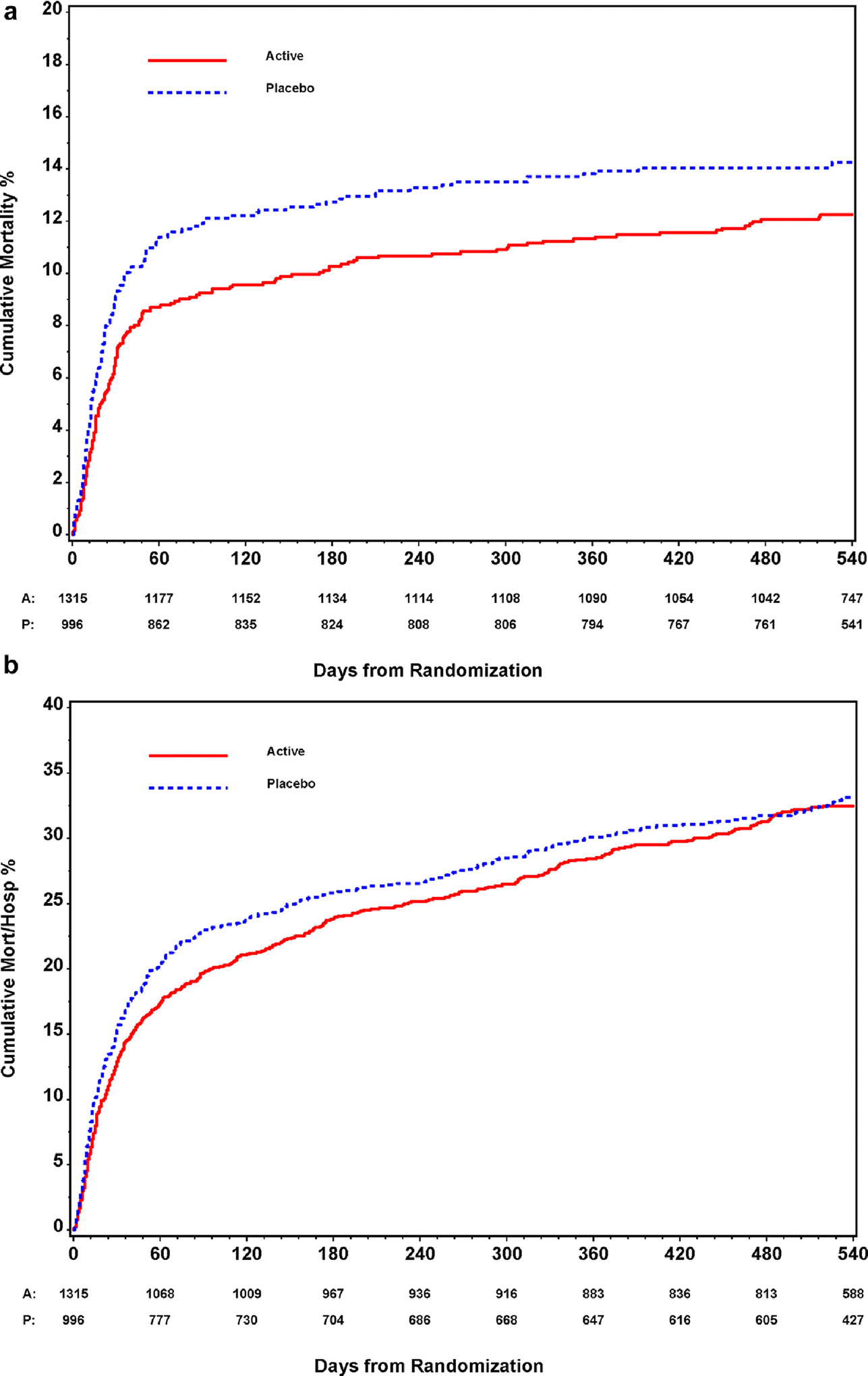
(a) Kaplan–Meier curve for mortality through month 18 for all included ACTIV-3/TICO trials. (b) Kaplan–Meier curve for mortality or rehospitalization through month 18 for all included ACTIV-3/TICO trials. In a pooled Cox-regression analysis of time to mortality through month 18 stratified by trial (a), there appeared to be an early, though not significant, benefit to passive immunotherapies after which the active agent and placebo curves neither diverge or converge in the long-term. Conversely, when incorporating rehospitalization (b), the possible early benefit is not maintained over time. ACTIV-3/TICO, Accelerating COVID-19 Therapeutic Interventions and Vaccines–Therapeutics for Inpatients with COVID-19.

**Fig. 3.**
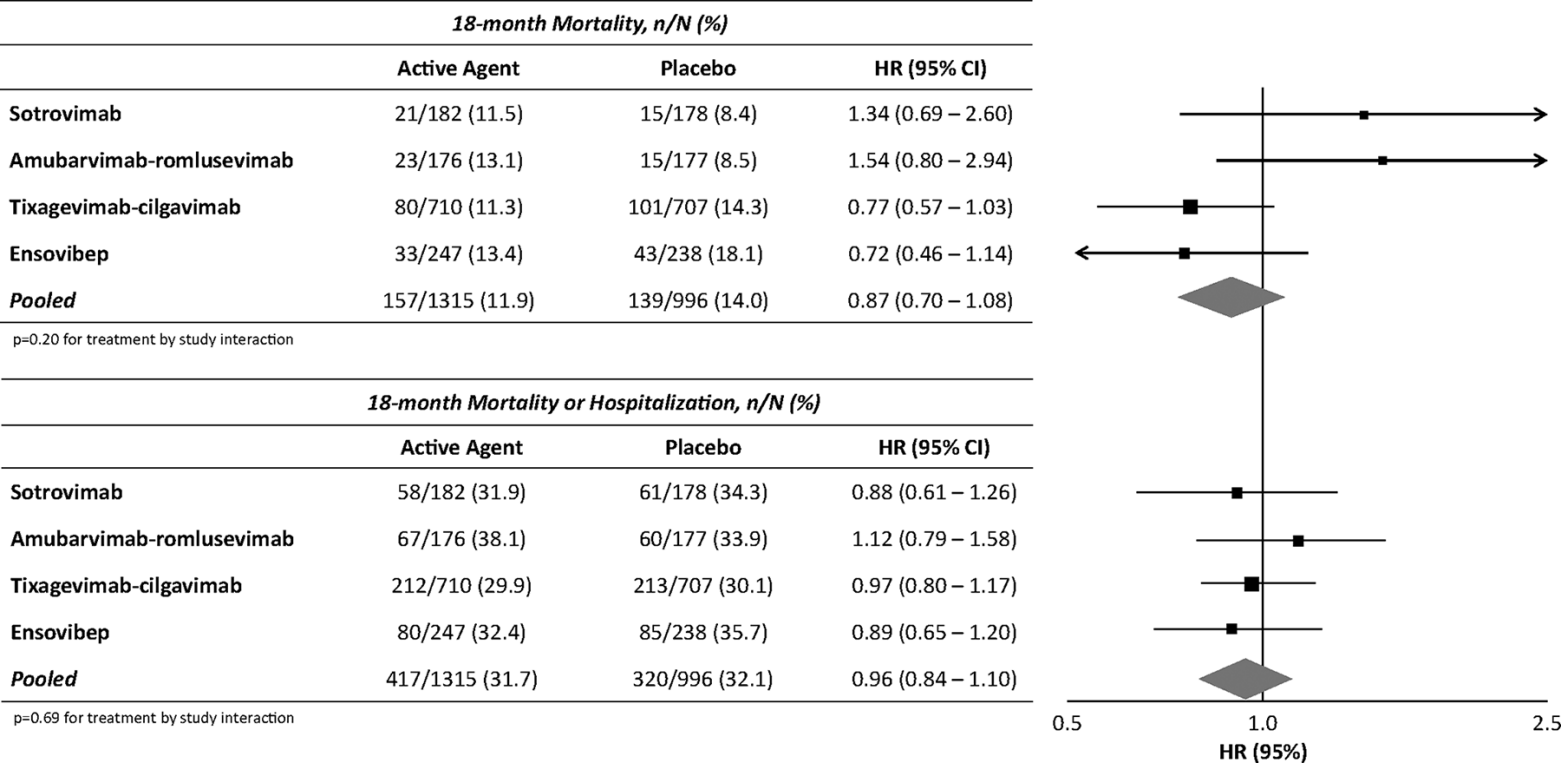
Cox-regression analysis comparing long-term outcomes between participants receiving active agents vs. placebo. HR, hazard ratio.

There was no significant difference in mortality across all active (11.9% [157/1315]) vs. placebo (14.0% [139/996]) arms of the study through 18 months of follow-up (hazard ratio [HR], 0.87; 95% CI, 0.70–1.08; p 0.22) (Figs. 2 and 3). Fig. S1 details the loss to follow-up between active and placebo groups, which was minimal through month 15 and similar between groups. Additionally, there was no significant difference in the treatment effect across the individual trials (p 0.20). Separating deaths into those occurring during the first 90 days from the time of enrolment and those occurring beyond day 90, there was also no significant difference in the treatment effect by time period (p 0.13 for interaction) (Table S4). Furthermore, there was no difference in the combined outcome of mortality or rehospitalization through month 18 across all active (31.7% [417/1315]) vs. placebo (32.1% [320/996]) arms of the study (HR, 0.96; 95% CI, 0.84–1.10; p 0.54) (Fig. 3). Similarly, there was not a significant difference in the treatment effect across trials (p 0.69). However, the hazard ratio comparing active to placebo for the composite endpoint did vary by time, with improvement through day 90 among patients receiving passive immunotherapy (HR, 0.84; 95% CI, 0.71–1.00), but no difference beyond day 90 through 18 months (HR, 1.22; 95% CI, 0.97–1.54) (p 0.012 for interaction) (Table S4).

These results remained consistent in sensitivity analyses excluding the largest trial (tixagevimab–cilgavimab) when comparing mortality (HR, 1.01; 95% CI, 0.73–1.38) and the composite of mortality and rehospitalization (HR, 0.95; 95% CI, 0.78–1.15) through month 18. However, the difference in the HR for mortality or hospitalization by time period was no longer significant when the tixagevimab–cilgavimab arm was excluded (p 0.65).

Among participants seronegative for anti-spike neutralizing antibodies at enrolment, there was not a significant difference between those receiving active agents vs. placebo in the rate of mortality (12.3% [74/600] vs. 15.5% [74/477]; HR, 0.78; 95% CI, 0.57–1.05) or in the rate of mortality or rehospitalization (32.5% [195/600] vs. 35.0% [167/477]; HR, 0.91; 95% CI, 0.75–1.10) through month 18 of follow-up. Hazard ratios were slightly higher among participants seropositive for anti-spike neutralizing antibodies when comparing mortality (HR, 1.11; 95% CI, 0.79–1.56) and mortality or rehospitalization (HR, 1.05; 95% CI, 0.86–1.28). The difference in the pooled treatment effect between anti-spike seronegative and seropositive participants was statistically significant for mortality (p 0.03 for interaction) but not for mortality or rehospitalization (p 0.19 for interaction) (Table 2). Fig. S2 details long-term mortality between active and placebo groups by SARS-CoV-2 anti-spike neutralizing antibody status at baseline. Fig. S3 details long-term mortality between active and placebo groups by SARS-CoV-2 anti-spike and anti-nucleocapsid antibody status at baseline.

## Discussion

In this pooled analysis of 18-month outcomes from our large, multinational, randomized placebo-controlled platform trial of long half-life passive immunotherapy for hospitalized patients with COVID-19, we did not identify any improvement in long-term outcomes nor harm associated with long half-life passive immunotherapy. Specifically, there were no sustained differences in mortality or combined mortality and rehospitalization between individuals receiving active agents vs. placebo, and differences in long-term outcomes between active and placebo groups did not differ by baseline serostatus.

Among the long half-life passive immunotherapies studied in the ACTIV-3/TICO master protocol, only tixagevimab–cilgavimab completed enrolment and was not terminated early for futility. Although there was no difference in the primary outcome of time to sustained recovery up to day 90 between participants receiving the tixagevimab–cilgavimab or placebo (relative risk reduction, 1.08; 95% CI, 0.97–1.20), mortality was lower for those receiving the active agent (HR, 0.70; 95% CI, 0.50–0.97) [3]. This trend was not seen with the other long half-life agents [2,4]. In our analysis of long-term mortality through 18 months from enrolment of all four agents, including tixagevimab–cilgavimab, there was no difference between participants who received active agents or placebo. This was also the case when using a composite outcome of mortality or rehospitalization through month 18. These findings were consistent in a sensitivity analysis that excluded the tixagevimab–cilgavimab trial, strongly suggesting that long half-life passive immunotherapies do not impact long-term mortality or serious morbidity.

Furthermore, although there appeared to be a possible signal for short-term harm in participants who were seropositive for anti-SARS-CoV-2 neutralizing antibodies at baseline receiving passive immunotherapies in some trials [2,5,11], we did not identify any impact of baseline serostatus on long-term treatment outcomes in this larger pooled analysis.

From the perspective of clinical trial design and implementation in a global pandemic, with competing interests and priorities for research staff, extending follow-up for these agents is a costly and time-consuming endeavour. Our data suggest that in future outbreaks, prolonged follow-up for an acute viral illness may not be justified. Additionally, rapid serological screening could be used to maximize enrolment of participants who are seronegative at baseline and most likely to benefit from passive immunotherapy.

### Limitations

Although this study is the largest of long-term outcomes of passive immunotherapy for COVID-19 in a clinical trial, it has some limitations. First, we did not measure neutralizing antibody titres beyond the first 90 days of follow-up, limiting our ability to comment on the durability of this immunity. We also did not account for post-enrolment interventions such as vaccination, or for recurrent COVID-19 episodes to determine whether passive immunity had any direct effects on immune response to vaccination or reinfection. Second, we did not define parameters for, nor directly measure, specific adverse effects in extended follow-up, such as non-fatal safety or efficacy events, other than what was captured by MedDRA-coded SOC for mortality or rehospitalization. Additionally, we were not sufficiently powered to assess these longer-term outcomes of individual agents. Lastly, although ACTIV-3/TICO was able to establish a large global network to conduct these clinical trials, there was limited enrolment from Asia and African regions.

## Conclusion

In our large study of long half-life passive immunotherapy for hospitalized patients with COVID-19, we did not find evidence of improved long-term outcomes or harm associated with these treatments. This suggests that in future outbreaks of acute viral infections, prolonging follow-up to three times the half-life of similar agents may introduce burdensome clinical trial procedures without appreciable benefit.

## Supplementary Material

1

2

## Figures and Tables

**Table 1:** Pooled (*N* = 2311) and individual ACTIV-3/TICO trial participant characteristics

Participant characteristics	Pooled	Sotrovimab	Amubarvimab-romlusevimab	Tixagevimab-cilgavimab	Ensovibep

**No. of participants, total (active agent)**	2311 (1315)	269 (182)	262 (176)	1351 (710)	429 (247)
**Age (y), median (IQR)**	57 (46–68)	60 (50–72)	60 (49–71)	55 (44–66)	57 (46–68)
**Sex, *n* (%)**					
**Male**	1337 (57.9)	156 (58.0)	149 (56.9)	785 (58.1)	247 (57.6)
**Female**	974 (42.1)	113 (42.0)	113 (43.1)	566 (41.9)	182 (42.4)
**Comorbid conditions, *n* (%)**					
**Obesity**^a^	1212 (52.6)	149 (55.4)	139 (53.1)	720 (53.6)	204 (47.7)
**Cardiovascular disease**	1085 (46.9)	154 (57.2)	156 (59.5)	592 (43.8)	183 (42.7)
**Diabetes**	650 (28.1)	102 (37.9)	88 (33.6)	357 (26.4)	103 (24.0)
**Chronic lung disease**	356 (15.4)	42 (15.6)	48 (18.3)	201 (14.9)	65 (15.2)
**Chronic kidney disease**	233 (10.1)	39 (14.5)	20 (7.6)	129 (9.5)	45 (10.5)
**Immunocompromised**	205 (8.9)	30 (11.2)	26 (9.9)	123 (9.1)	26 (6.1)
**Geographical region, *n* (%)**					
**North America**	1784 (77.2)	253 (94.1)	250 (95.4)	989 (73.2)	292 (68.1)
**Europe**	364 (15.8)	16 (5.9)	12 (4.6)	255 (18.9)	81 (18.9)
**Africa**	122 (5.3)	0	0	87 (6.4)	35 (8.2)
**Asia**	41 (1.8)	0	0	20 (1.5)	21 (4.9)
**SARS-CoV-2 vaccination status, *n* (%)**					
**Unvaccinated**	1784 (77.2)	251 (93.3)	246 (93.9)	991 (73.4)	296 (69.0)
**Fully vaccinated**^b^	302 (13.1)	1 (0.4)	0	190 (14.1)	111 (25.9)
**Partially vaccinated**^c^	225 (9.7)	17 (6.3)	16 (6.1)	170 (12.6)	22 (5.1)
**Serostatus, *n* (%)**					
**Anti-SARS-CoV-2 nucleocapsid positive**	1403 (62.8)	154 (59.0)	162 (63.3)	825 (63.3)	262 (63.1)
**Anti-SARS-CoV-2 spike positive**	1157 (51.8)	106 (40.6)	109 (42.6)	695 (53.4)	247 (59.5)
**SARS-CoV-2 variant, *n* (%)**					
**Delta**	986 (43.6)	0	1 (0.4)	642 (48.7)	343 (82.7)
**Non-Delta**	1278 (56.4)	269 (100)	261 (99.6)	676 (51.3)	72 (17.3)
**Days since symptom onset, median (IQR)**	8 (6–10)	8 (5–9)	8 (5–9)	8 (6–10)	8 (5–9)
**Supplemental oxygen status, *n* (%)**					
**Not receiving supplemental oxygen**	570 (24.7)	91 (33.8)	83 (31.7)	317 (23.5)	79 (18.4)
**Supplemental oxygen <4 L/min**	835 (36.1)	119 (44.2)	108 (41.2)	484 (35.8)	124 (28.9)
**Supplemental oxygen ≥4 L/min**	668 (28.9)	59 (21.9)	71 (27.1)	401 (29.7)	137 (31.9)
**NIPPV or HFHHNC**	238 (10.3)	0	0	149 (11.0)	89 (20.7)
**Pre-enrolment therapeutics, *n* (%)**					
**Remdesivir**	2129 (92.1)	244 (90.7)	234 (89.3)	1247 (92.3)	404 (94.2)
**Corticosteroids**	1627 (70.4)	170 (63.2)	159 (60.7)	985 (72.9)	313 (73.0)

ACTIV-3/TICO, Accelerating COVID-19Therapeutic Interventions and Vaccines—Therapeutics for Inpatients with COVID-19; BMI, body mass index; HFHHNC, high-flow high-humidity nasal canula; IQR, interquartile range; NIPPV, non-invasive positive pressure ventilation.

a BMI ≥30 kg/m^2^.

b Definition of fully vaccinated: two-dose course completed, and symptoms started >14 days after the last dose.

c Definition of partially vaccinated: two-dose course completed, and symptoms started ≤14 days after the last dose; or only one dose received.

**Table 2:** Cox-regression summary of long-term outcomes between participants receiving active agents vs. placebo stratified by serostatus (anti-spike neutralizing antibodies) at baseline

ACTIV-3/TICO trial	18-month mortality, *n/N* (%)					
	Baseline seronegative			Baseline seropositive		
	Active agent	Placebo	HR (95% CI)	Active agent	Placebo	HR (95% CI)

Sotrovimab	16/106 (15.1)	12/97 (12.4)	1.16 (0.55–2.46)	5/71 (7.0)	2/77 (2.6)	2.75 (0.53–14.16)
Amubarvimab–romlusevimab	14/101 (13.9)	12/96 (12.5)	1.08 (0.50–2.33)	9/70 (12.9)	2/77 (2.6)	5.08 (1.10–23.53)
Tixagevimab–cilgavimab	36/307 (11.7)	54/337 (16.0)	0.72 (0.47–1.10)	38/382 (9.9)	37/339 (10.9)	0.89 (0.57–1.40)
Ensovibep	8/86 (9.3)	21/109 (19.3)	0.45 (0.20–1.02)	23/154 (14.9)	18/118 (15.3)	0.99 (0.53–1.83)
Pooled	74/600 (12.3)	74/477 (15.5)	0.78 (0.57–1.05)	75/677 (11.1)	53/480 (11.0)	1.11 (0.79–1.56)

	18-month mortality or hospitalization, *n/N* (%)					

Sotrovimab	38/106 (35.8)	35/97 (36.1)	0.91 (0.58–1.45)	18/71 (25.4)	24/77 (31.2)	0.80 (0.43–1.47)
Amubarvimab–romlusevimab	39/101 (38.6)	34/96 (35.4)	1.05 (0.66–1.66)	27/70 (38.6)	24/77 (31.2)	1.31 (0.75–2.27)
Tixagevimab–cilgavimab	94/307 (30.6)	112/337 (33.2)	0.90 (0.68–1.19)	107/382 (28.0)	86/339 (25.4)	1.07 (0.81–1.42)
Ensovibep	24/86 (27.9)	40/109 (36.7)	0.72 (0.44–1.20)	52/154 (33.8)	39/118 (33.1)	1.03 (0.68–1.55)
Pooled	195/600 (32.5)	167/477 (35.0)	0.91 (0.75–1.10)	204/677 (30.1)	133/480 (27.7)	1.05 (0.86–1.28)

p 0.03 for treatment by serostatus interaction–mortality; p 0.19 for treatment by serostatus interaction–mortality or hospitalization.
