# Supplementary material for: Long-term outcomes of passive immunotherapy for COVID-19: a pooled analysis of a large multinational platform randomized clinical trial
Source: Clin Microbiol Infect. Author manuscript; Available in PMC 2025 Jun 1. (PMC12068974; doi:10.1016/j.cmi.2025.02.002)

Supplementary Appendix

1. Participant enrollments per country Page 2
2. Participant deaths (through month 18) Page 3
3. Participant rehospitalizations (day 90 through month 18) Page 4
4. Long-term outcomes between groups, by time Page 5
5. Loss to follow-up (through month 18) Page 6
6. Kaplan meier curve of long-term outcomes between groups, by serostatus Page 7
7. Long-term outcomes between groups, by serostatus (detailed) Page 8

Supplementary Table 1. Number of participants enrolled in ACTIV-3/TICO per country.

Country	Number of participants enrolled, n (%)
United States	1784 (77.2)
Spain	145 (6.3)
Uganda	122 (5.3)
Greece	84 (3.6)
Denmark	54 (2.3)
Singapore	41 (1.8)
United Kingdom	31 (1.3)
Switzerland	24 (1.0)
Poland	17 (0.7)
Nigeria	9 (0.4)

Supplementary Table 2. Participant deaths through month 18 by Medical Dictionary for Regulatory Activities (MedDRA) – System Organ Class (SOC).

MedDRA – SOC	Active (n=1315), n (%)	Placebo (n=996), n (%)	Total (N=2311), n (%)
Blood and lymphatic system	0	0	0
Cardiac	12 (0.9)	11 (1.1)	23 (1.0)
Congenital, familial, genetic	0	0	0
Ear and labyrinth	0	0	0
Endocrine	0	0	0
Eye	0	0	0
Gastrointestinal	0	1 (0.1)	1 (0.0)
General and administration site	15 (1.1)	6 (0.6)	21 (0.9)
Hepatobiliary	0	2 (0.2)	2 (0.1)
Immune system	0	0	0
Infections and infestations	44 (3.3)	42 (4.2)	86 (3.7)
Injury, poisoning, procedural	3 (0.2)	2 (0.2)	5 (0.2)
Investigations	0	0	0
Metabolism and nutrition	3 (0.2)	0	4 (0.2)
Musculoskeletal, connective tissue	0	0	0
Neoplasms – benign and malignant	12 (0.9)	6 (0.6)	18 (0.8)
Nervous system	2 (0.2)	3 (0.3)	5 (0.2)
Pregnancy, puerperium, perinatal	0	0	0
Product issues	0	0	0
Psychiatric	0	0	0
Renal and urinary	2 (0.2)	0	2 (0.1)
Reproductive system and breast	0	0	0
Respiratory, thoracic, mediastinal	62 (4.7)	64 (6.4)	126 (5.5)
Skin and subcutaneous tissue	0	0	0
Social circumstances	0	0	0
Surgical and medical procedures	0	0	0
Vascular	2 (0.2)	1 (0.1)	3 (0.1)
**Total deaths**	157 (11.9)	139 (14.0)	296 (12.3)

Supplementary Table 3. Participant rehospitalizations between day 90 through month 18 by Medical Dictionary for Regulatory Activities (MedDRA) – System Organ Class (SOC).

MedDRA – SOC	Active, n (%)	Placebo, n (%)	Total, n (%)
Blood and lymphatic system	12 (3.1)	3 (1.3)	15 (2.5)
Cardiac	38 (10.0)	30 (13.2)	68 (11.2)
Congenital, familial, genetic	1 (0.3)	0	1 (0.2)
Ear and labyrinth	0	0	0
Endocrine	1 (0.3)	2 (0.9)	3 (0.5)
Eye	3 (0.8)	1 (0.4)	4 (0.7)
Gastrointestinal	24 (6.3)	16 (7.0)	40 (6.6)
General and administration site	9 (2.4)	6 (2.6)	15 (2.5)
Hepatobiliary	11 (2.9)	4 (1.8)	15 (2.5)
Immune system	4 (1.0)	1 (0.4)	5 (0.8)
Infections and infestations	70 (18.4)	53 (23.3)	123 (20.2)
Injury, poisoning, procedural	21 (5.5)	11 (4.8)	32 (5.3)
Investigations	0	0	0
Metabolism and nutrition	26 (6.8)	12 (5.3)	38 (6.3)
Musculoskeletal, connective tissue	17 (4.5)	9 (4.0)	26 (4.3)
Neoplasms – benign and malignant	9 (2.4)	6 (2.6)	15 (2.5)
Nervous system	26 (6.8)	22 (9.7)	48 (7.9)
Pregnancy, puerperium, perinatal	1 (0.3)	2 (0.9)	3 (0.5)
Product issues	1 (0.3)	0	1 (0.2)
Psychiatric	7 (1.8)	3 (1.3)	10 (1.6)
Renal and urinary	19 (5.0)	10 (4.4)	29 (4.8)
Reproductive system and breast	4 (1.0)	1 (0.4)	5 (0.8)
Respiratory, thoracic, mediastinal	54 (14.2)	29 (12.8)	83 (13.7)
Skin and subcutaneous tissue	1 (0.3)	0	1 (0.2)
Social circumstances	0	0	0
Surgical and medical procedures	5 (1.3	3 (1.3)	8 (1.3)
Vascular	17 (4.5)	3 (1.3)	20 (3.3)
**Total rehospitalizations**	381	227	608

Supplementary Table 4. Cox-regression summary of long-term outcomes between groups receiving active agents versus placebo by time.

	18-month Mortality, n/N (%)	
	**HR (95% CI), first 90 days**	**HR (95% CI), beyond day 90**
**Sotrovimab**	1.03 (0.49 – 2.20)	3.35 (0.70 – 16.11)
**Amubarvimab-romlusevimab**	1.16 (0.55 – 2.45)	3.91 (0.83 – 18.43)
**Tixagevimab-cilgavimab**	0.69 (0.50 – 0.96)	1.21 (0.62 – 2.38)
**Ensovibep**	0.81 (0.50 – 1.32)	0.34 (0.09 – 1.28)
Pooled	0.79 (0.62 – 1.01)	1.30 (0.79 – 2.14)
Ratio of HRs	1.55 (0.88 – 2.71), p=0.127	
	**18-month Mortality or Hospitalization, n/N (%)**	
	**HR (95% CI), first 90 days**	**HR (95% CI), beyond day 90**
**Sotrovimab**	0.79 (0.50 – 1.23)	1.08 (0.58 – 1.99)
**Amubarvimab-romlusevimab**	1.02 (0.66 – 1.56)	1.34 (0.74 – 2.43)
**Tixagevimab-cilgavimab**	0.78 (0.62 – 0.99)	1.43 (1.03 – 1.98)
**Ensovibep**	0.92 (0.64 – 1.34)	0.82 (0.48 – 1.40)
Pooled	0.84 (0.71 – 1.00)	1.22 (0.97 – 1.54)
Ratio of HRs	1.45 (1.08 – 1.93), p=0.012	

Supplementary Figure 1. Kaplan Meier curve of loss to follow-up for groups receiving active agents and placebo through month 18 for all included ACTIV-3/TICO trials.

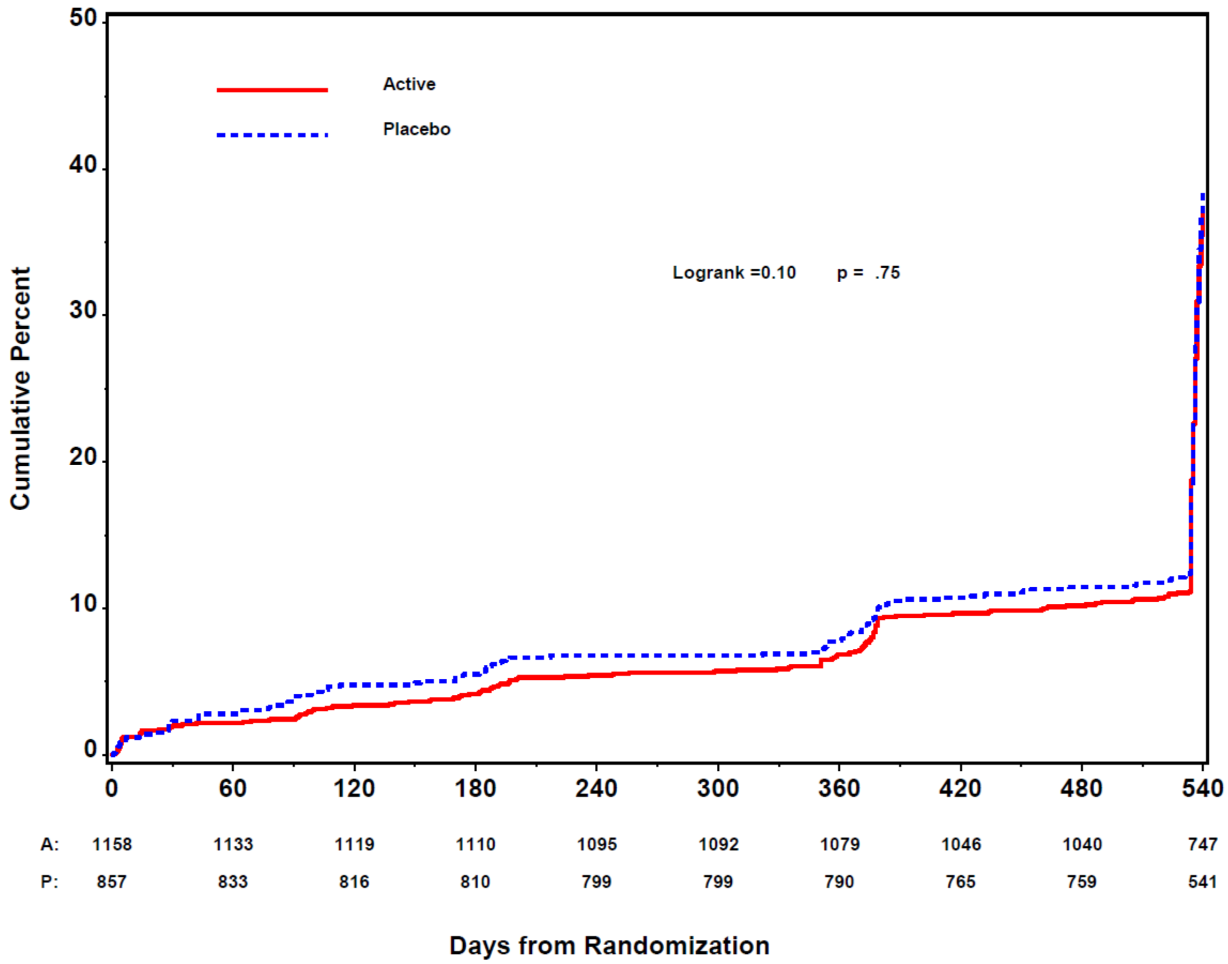

Supplementary Figure 2. Kaplan Meier curve for long-term mortality between active and placebo groups by SARS-CoV-2 anti-spike neutralizing antibody status at baseline.

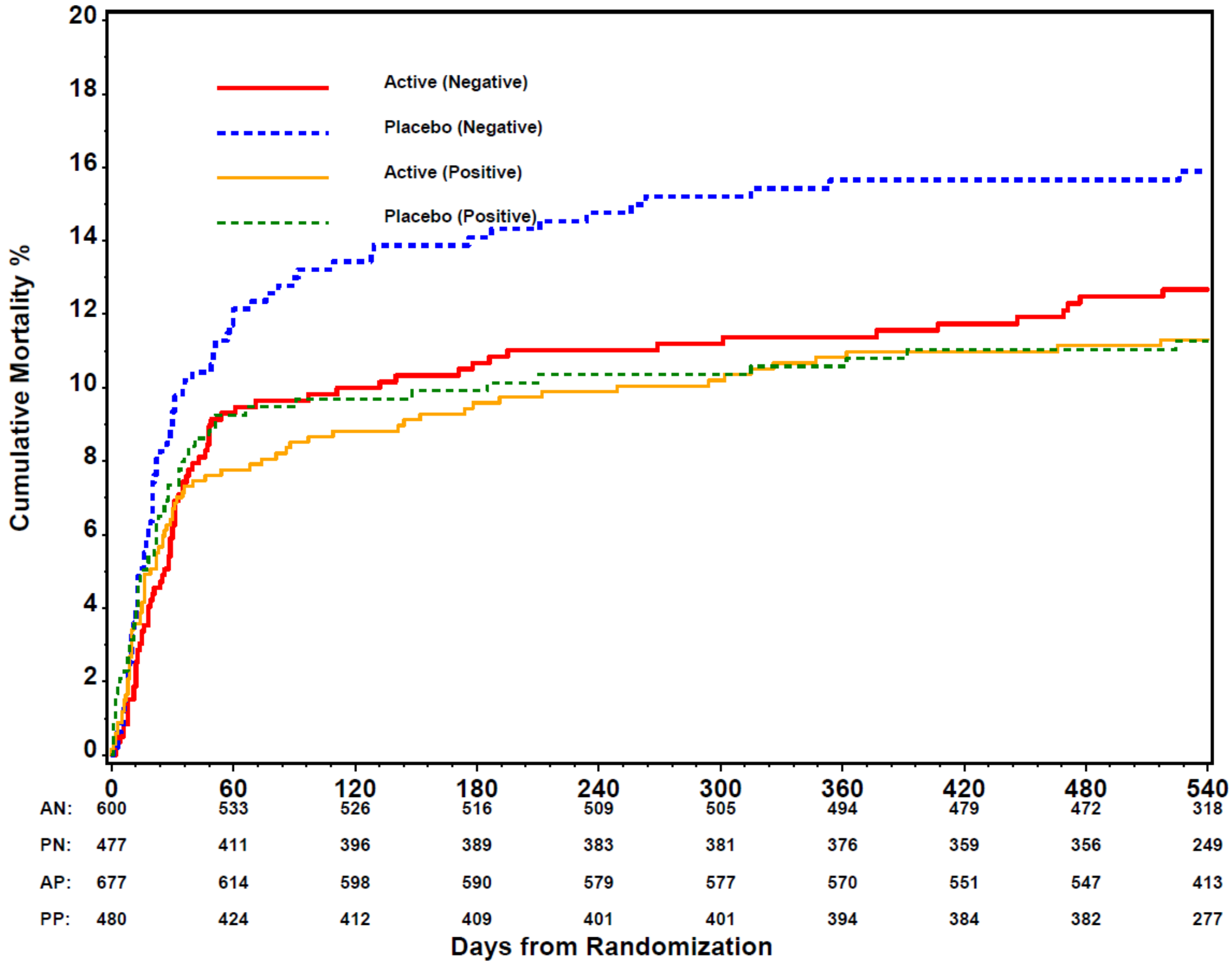

Supplementary Figure 3. Cox regression analyses comparing long-term mortality between active and placebo groups by SARS-CoV-2 anti-spike and anti-nucleocapsid antibody status at baseline.

Baseline Serostatus		*18-month Mortality, n/N (%)*		
**Anti-Spike**	**Anti-Nucleocapsid**	**Active Agent**	**Placebo**	**HR (95% CI)**
Negative	Negative	50/354 (14.1%)	45/245 (18.4%)	0.74 (0.51 – 1.08)
Negative	Positive	24/246 (9.8%)	29/232 (12.5%)	0.80 (0.48 – 1.34)
Positive	Negative	13/142 (9.1%)	10/91 (11.0%)	0.97 (0.43 – 2.16)
Positive	Positive	62/535 (11.6%)	43/389 (11.1%)	1.15 (0.79 – 1.68)

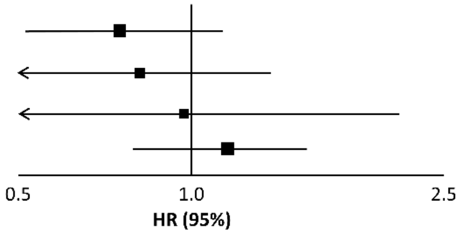

Supplement: 1 [file NIHMS2060123-supplement-1.pdf]
