# Supplementary material for: Long-term outcomes of passive immunotherapy for COVID-19: a pooled analysis of a large multinational platform randomized clinical trial
Source: Clin Microbiol Infect. Author manuscript; Available in PMC 2025 Jun 1. (PMC12068974; doi:10.1016/j.cmi.2025.02.002)
Supplement: 2 [file NIHMS2060123-supplement-2.pdf]

Supplementary Appendix 2

List of Study Personnel and Affiliations

	Last name	First name	Degrees	Affiliation
NIH	Sahner	David	M.D.	U.S. National Institute of Allergy and Infectious Diseases incl. Department of Clinical Research
	Tierney	John	B.Sc.N.	U.S. National Institute of Allergy and Infectious Diseases incl. Department of Clinical Research
	Vogel	Susan E.	R.N., B.Sc.N.	U.S. National Institute of Allergy and Infectious Diseases incl. Department of Clinical Research
	Herpin	Betsey R.	M.Sc.N., C.C.R.C., R.N.	U.S. National Institute of Allergy and Infectious Diseases incl. Department of Clinical Research
	Smolskis	Mary C.	B.Sc.N., M.A	U.S. National Institute of Allergy and Infectious Diseases incl. Department of Clinical Research
	McKay	Laura A.	M.SC.	U.S. National Institute of Allergy and Infectious Diseases incl. Department of Clinical Research
	Cahill	Kelly	R.N., M.Sc., C.C.R.C., R.A.C.	U.S. National Institute of Allergy and Infectious Diseases incl. Department of Clinical Research
	Crew	Page	PharmD., M.P.H., B.C.P.S.	U.S. National Institute of Allergy and Infectious Diseases incl. Department of Clinical Research
	Sardana	Ratna	B.A.	U.S. National Institute of Allergy and Infectious Diseases incl. Department of Clinical Research
	Raim	Sharon Segal	M.P.H.	U.S. National Institute of Allergy and Infectious Diseases incl. Department of Clinical Research
	Hensely	Lisa	Ph.D.	U.S. National Institute of Allergy and Infectious Diseases incl. Department of Clinical Research
	Lorenzo	Johsua	M.P.H.	U.S. National Institute of Allergy and Infectious Diseases incl. Department of Clinical Research
	Mock	Rebecca	Ph.D., R.A.C.	U.S. National Institute of Allergy and Infectious Diseases incl. Department of Clinical Research
	Zuckerman	Judith	B.S.N.	U.S. National Institute of Allergy and Infectious Diseases incl. Department of Clinical Research
	Atri	Negin	M.P.H.	U.S. National Institute of Allergy and Infectious Diseases incl. Department of Clinical Research
	Miller	Mark	PharmD., B.C.P.S., R.A.C.	U.S. National Institute of Allergy and Infectious Diseases incl. Department of Clinical Research
	Vallee	David	PharmD., M.P.H.	U.S. National Institute of Allergy and Infectious Diseases incl. Department of Clinical Research
	Chung	Lucy	PharmD., C.C.R.P.	U.S. National Institute of Allergy and Infectious Diseases incl. Department of Clinical Research
	Kang	Nayon	PharmD., M.S.	U.S. National Institute of Allergy and Infectious Diseases incl. Department of Clinical Research
	Barrett	Kevin	R.N., B.Sc.N.	U.S. National Institute of Allergy and Infectious Diseases incl. Department of Clinical Research
FNIH	Adam	Stacey J.	Ph.D.	Foundation for the National Institutes of Health, The Accelerating COVID-19 Therapeutic Interventions and Vaccines (ACTIV) and Operation Warp Speed
	Read	Sarah	M.D.	Foundation for the National Institutes of Health, The Accelerating COVID-19 Therapeutic Interventions and Vaccines (ACTIV) and Operation Warp Speed
	Draghia-Akli	Ruxandra	M.D., Ph.D.	Foundation for the National Institutes of Health, The Accelerating COVID-19 Therapeutic Interventions and Vaccines (ACTIV) and Operation Warp Speed

	Currier	Judy	M.D.	Foundation for the National Institutes of Health, The Accelerating COVID-19 Therapeutic Interventions and Vaccines (ACTIV) and Operation Warp Speed
	Hughes	Eric	M.D., Ph.D.	Foundation for the National Institutes of Health, The Accelerating COVID-19 Therapeutic Interventions and Vaccines (ACTIV) and Operation Warp Speed
	Harrigan	Rachel H.	M.D.	Foundation for the National Institutes of Health, The Accelerating COVID-19 Therapeutic Interventions and Vaccines (ACTIV) and Operation Warp Speed
**SDMC**	Amos	Laura		INSIGHT SDMC, Division of Biostatistics, School of Public Health and School of Statistics, University of Minnesota, Minneapolis, MN, USA
	Carlsen	Amy	R.N.	INSIGHT SDMC, Division of Biostatistics, School of Public Health and School of Statistics, University of Minnesota, Minneapolis, MN, USA
	Carter	Anita		INSIGHT SDMC, Division of Biostatistics, School of Public Health and School of Statistics, University of Minnesota, Minneapolis, MN, USA
	Collins	Gary	M.S.	INSIGHT SDMC, Division of Biostatistics, School of Public Health and School of Statistics, University of Minnesota, Minneapolis, MN, USA
	Davis	Bionca	M.P.H.	INSIGHT SDMC, Division of Biostatistics, School of Public Health and School of Statistics, University of Minnesota, Minneapolis, MN, USA
	Denning	Eileen	M.P.H.	INSIGHT SDMC, Division of Biostatistics, School of Public Health and School of Statistics, University of Minnesota, Minneapolis, MN, USA
	DuChene	Alain		INSIGHT SDMC, Division of Biostatistics, School of Public Health and School of Statistics, University of Minnesota, Minneapolis, MN, USA
	Eckroth	Kate	M.P.H.	INSIGHT SDMC, Division of Biostatistics, School of Public Health and School of Statistics, University of Minnesota, Minneapolis, MN, USA
	Engen	Nicole	M.S.	INSIGHT SDMC, Division of Biostatistics, School of Public Health and School of Statistics, University of Minnesota, Minneapolis, MN, USA
	Frase	Alex		INSIGHT SDMC, Division of Biostatistics, School of Public Health and School of Statistics, University of Minnesota, Minneapolis, MN, USA
	Gandits	Greg	M.S.	INSIGHT SDMC, Division of Biostatistics, School of Public Health and School of Statistics, University of Minnesota, Minneapolis, MN, USA
	Grund	Birgit	Ph.D.	INSIGHT SDMC, Division of Biostatistics, School of Public Health and School of Statistics, University of Minnesota, Minneapolis, MN, USA
	Harrison	Merrie		INSIGHT SDMC, Division of Biostatistics, School of Public Health and School of Statistics, University of Minnesota, Minneapolis, MN, USA
	Hurlbut	Nancy		INSIGHT SDMC, Division of Biostatistics, School of Public Health and School of Statistics, University of Minnesota, Minneapolis, MN, USA
	Kaiser	Payton		INSIGHT SDMC, Division of Biostatistics, School of Public Health and School of Statistics, University of Minnesota, Minneapolis, MN, USA
	Koopmeiners	Joseph	Ph.D.	INSIGHT SDMC, Division of Biostatistics, School of Public Health and School of Statistics, University of Minnesota, Minneapolis, MN, USA
	Larson	Gregg	M.A.	INSIGHT SDMC, Division of Biostatistics, School of Public Health and School of Statistics, University of Minnesota, Minneapolis, MN, USA

	Meger	Sue		INSIGHT SDMC, Division of Biostatistics, School of Public Health and School of Statistics, University of Minnesota, Minneapolis, MN, USA
	Mistry	Shweta Sharma	M.S.	INSIGHT SDMC, Division of Biostatistics, School of Public Health and School of Statistics, University of Minnesota, Minneapolis, MN, USA
	Murray	Thomas	Ph.D.	INSIGHT SDMC, Division of Biostatistics, School of Public Health and School of Statistics, University of Minnesota, Minneapolis, MN, USA
	Nelson	Ray	R.N.	INSIGHT SDMC, Division of Biostatistics, School of Public Health and School of Statistics, University of Minnesota, Minneapolis, MN, USA
	Quan	Kien	M.S.	INSIGHT SDMC, Division of Biostatistics, School of Public Health and School of Statistics, University of Minnesota, Minneapolis, MN, USA
	Quan	Siu Fun		INSIGHT SDMC, Division of Biostatistics, School of Public Health and School of Statistics, University of Minnesota, Minneapolis, MN, USA
	Reilly	Cavan	Ph.D.	INSIGHT SDMC, Division of Biostatistics, School of Public Health and School of Statistics, University of Minnesota, Minneapolis, MN, USA
	Siegel	Lianne	Ph.D.	INSIGHT SDMC, Division of Biostatistics, School of Public Health and School of Statistics, University of Minnesota, Minneapolis, MN, USA
	Thompson	Greg		INSIGHT SDMC, Division of Biostatistics, School of Public Health and School of Statistics, University of Minnesota, Minneapolis, MN, USA
	Vock	David	Ph.D.	INSIGHT SDMC, Division of Biostatistics, School of Public Health and School of Statistics, University of Minnesota, Minneapolis, MN, USA
	Walski	Jamie	M.H.A.	INSIGHT SDMC, Division of Biostatistics, School of Public Health and School of Statistics, University of Minnesota, Minneapolis, MN, USA
**CTSN**	Gelijns	Annetine C.	Ph.D.	Cardiothoracic Surgical Trials Network (CTSN) International Coordinating Center (ICC). Icahn School of Medicine at Mount Sinai, New York, USA
	Moskowitz	Alan J.	M.D.	Cardiothoracic Surgical Trials Network (CTSN) International Coordinating Center (ICC). Icahn School of Medicine at Mount Sinai, New York, USA
	Bagiella	Emilia	Ph.D.	Cardiothoracic Surgical Trials Network (CTSN) International Coordinating Center (ICC). Icahn School of Medicine at Mount Sinai, New York, USA
	Moquete	Ellen	R.N., B.S.N.	Cardiothoracic Surgical Trials Network (CTSN) International Coordinating Center (ICC). Icahn School of Medicine at Mount Sinai, New York, USA
	O'Sullivan	Karen	M.P.H.	Cardiothoracic Surgical Trials Network (CTSN) International Coordinating Center (ICC). Icahn School of Medicine at Mount Sinai, New York, USA
	Marks	Mary E.	R.N., B.S.N.	Cardiothoracic Surgical Trials Network (CTSN) International Coordinating Center (ICC). Icahn School of Medicine at Mount Sinai, New York, USA
	Accardi	Evan	B.A.	Cardiothoracic Surgical Trials Network (CTSN) International Coordinating Center (ICC). Icahn School of Medicine at Mount Sinai, New York, USA
	Kinzel	Emily	M.P.H.	Cardiothoracic Surgical Trials Network (CTSN) International Coordinating Center (ICC). Icahn School of Medicine at Mount Sinai, New York, USA
	Burris	Sarah	M.H.A.	Cardiothoracic Surgical Trials Network (CTSN) International Coordinating Center (ICC). Icahn School of Medicine at Mount Sinai, New York, USA

Bedoya	Gabriela	B.S:	Cardiothoracic Surgical Trials Network (CTSN) International Coordinating Center (ICC). Icahn School of Medicine at Mount Sinai, New York, USA
Gupta	Lola	M.P.H.	Cardiothoracic Surgical Trials Network (CTSN) International Coordinating Center (ICC). Icahn School of Medicine at Mount Sinai, New York, USA
Overbey	Jessica R.	Dr.P.H.	Cardiothoracic Surgical Trials Network (CTSN) International Coordinating Center (ICC). Icahn School of Medicine at Mount Sinai, New York, USA
Santos	Milerva	M.P.A.	Cardiothoracic Surgical Trials Network (CTSN) International Coordinating Center (ICC). Icahn School of Medicine at Mount Sinai, New York, USA
Gillinov	Marc A.	M.D.	CTSN Steering Committee Chair, Cleveland Clinic Foundation
Miller	Marissa A.	D.V.M, M.P.H.	U.S. National Heart Lung and Blood Institute
Taddei-Peters	Wendy C.	Ph.D.	U.S. National Heart Lung and Blood Institute
Fenton	Kathleen	M.D., M.S.	U.S. National Heart Lung and Blood Institute
Sandkovsky	Uriel	M.D., M.S.	Baylor, Scott and White Health
Gottlieb	Robert L.	M.D., Ph.D	Baylor, Scott and White Health
Mack	Michael	M.D.	Baylor, Scott and White Health
Berhe	Mezgebe	M.D., M.P.H.	Baylor, Scott and White Health
Haley	Clinton	M.D., M.P.H.	Baylor, Scott and White Health
Dishner	Emma	M.D., M.P.H.	Baylor, Scott and White Health
Bettacchi	Christopher	M.D.	Baylor, Scott and White Health
Golden	Kevin	M.D.	Baylor, Scott and White Health
Duhaime	Erin	P.A.-C.	Baylor, Scott and White Health
Ryan	Madison	B.S.	Baylor, Scott and White Health
Burris	Sarah	M.H.A.	Baylor, Scott and White Health
Tallmadge	Catherine	B.A., C.M.A.	Baylor, Scott and White Health
Estrada	Lorie	C.C.R.C.	Baylor, Scott and White Health
Jones	Felecia	CRC	Baylor, Scott and White Health
Villa	Samatha		Baylor, Scott and White Health
Wang	Samatha	BSN, RN	Baylor, Scott and White Health
Robert	Raven	MPH	Baylor, Scott and White Health
Coleman	Tanquinisha		Baylor, Scott and White Health
Clariday	Laura		Baylor, Scott and White Health
Baker	Rebecca	BSN. RN	Baylor, Scott and White Health
Hurutado-Rodriguez	Mariana	BSN, CCRC, CPXP, CMI	Baylor, Scott and White Health
Iram	Nazia	CCRC	Baylor, Scott and White Health
Fresnedo	Michelle		Baylor, Scott and White Health
Davis	Allyson		Baylor, Scott and White Health
Leonard	Kiara		Baylor, Scott and White Health
Ramierez	Noelia		Baylor, Scott and White Health
Thammavong	Jon	B.S.	Baylor, Scott and White Health
Duque	Krizia		Baylor, Scott and White Health
Turner	Emma		Baylor, Scott and White Health
Fisher	Tammy	M.B.A, M.S.N, R.N, C.C.R.C.	Baylor, Scott and White Health
Robinson	Dianna	LVN, CCRC	Baylor, Scott and White Health

Ransom	Desirae	MS, CCRC	Baylor, Scott and White Health
Maldonado	Nicholas	B.A., A.C.R.P.,-CP	Baylor, Scott and White Health
Lusk	Erica	CCRP	Baylor, Scott and White Health
Killian	Aaron	PharmD.	Baylor, Scott and White Health
Palacious	Adriana	PharmD.	Baylor, Scott and White Health
Solis	Edilia	BS, CPhT	Baylor, Scott and White Health
Jerrow	Janet		Baylor, Scott and White Health
Watts	Matthew		Baylor, Scott and White Health
Whitacre	Heather		Baylor, Scott and White Health
Cothran	Elizabeth		Baylor, Scott and White Health
Smith	Peter K.	M.D.	Duke University Health System
Barkauskas	Christina E.	M.D.	Duke University Health System
Vekstein	Andrew M.	M.D.	Duke University Health System
Ko	Emily R.	M.D., PhD.	Duke University Health System
Dreyer	Grace R.	PA-C	Duke University Health System
Stafford	Neil	M.D.	Duke University Health System
Brooks	Megan	M.D.	Duke University Health System
Der	Tatyana	M.D.	Duke University Health System
Witte	Marie	M.D.	Duke University Health System
Gamarallage	Ruwan	M.D.	Duke University Health System
Franzone	John	M.D.	Duke University Health System
Ivey	Noel	M.D.	Duke University Health System
Lumsden	Rebecca H.	M.D.	Duke University Health System
Mosaly	Nilima	M.D.	Duke University Health System
Mourad	Ahmaad	M.D.	Duke University Health System
Holland	Thomas L.	M.D.	Duke University Health System
Motta	Mary	B.Sc.N., R.N.	Duke University Health System
Lane	Kathleen	B.Sc.N., R.N.	Duke University Health System
McGowan	Lauren M.	B.Sc., E.M.T.B.	Duke University Health System
Stout	Jennifer	B.Sc.	Duke University Health System
Aloor	Heather	M.P.H., M.S.	Duke University Health System
Bragg	Kennesha M.	M.S.	Duke University Health System
Toledo	Barvina	M.A.	Duke University Health System
McLendon-Arvik	Beth	PharmD.	Duke University Health System
Bussadori	Barbara	R.P.h.	Duke University Health System
Hollister	Beth A.	B.Sc.N., R.N	Duke University Health System
Griffin	Michelle	M.P.H., E.M.T.P.	Duke University Health System
Giangiacomo	Dana M.		Duke University Health System
Rodriguez	Vicente	M.D.	Lutheran Medical Group
Bokhart	Gordon	PharmD.	Lutheran Medical Group
Eichman	Sharon M.		Lutheran Medical Group
Parrino	Patrick E.	M.D., F.A.C.S.	Ochsner Clinic
Spindel	Stephen	M.D.	Ochsner Clinic
Bansal	Aditya	M.D.	Ochsner Clinic
Baumgarten	Katherine	M.D., F.A.C.P., F.I.D.S.A.	Ochsner Clinic
Hand	Johnathan	M.D.	Ochsner Clinic

Vonderhaar	Derek	M.D.	Ochsner Clinic
Nossaman	Bobby	M.D.	Ochsner Clinic
Laudun	Sylvia	D.N.P., M.B.A., R.N., C.P.H.Q.	Ochsner Clinic
Ames	DeAnna	M.S.	Ochsner Clinic
Broussard	Shane		Ochsner Clinic
Hernandez	Nilmo		Ochsner Clinic
Isaac	Geralyn	PharmD.	Ochsner Clinic
Dinh	Huan	PharmD.	Ochsner Clinic
Zheng	Yiling	PharmD.	Ochsner Clinic
Tran	Sonny	PharmD.	Ochsner Clinic
McDaniel	Hunter		Ochsner Clinic
Crovetto	Nicolle	M.S.	Ochsner Clinic
Perin	Emerson	M.D., PhD.	Texas Heart Institute
Costello	Briana	M.D.	Texas Heart Institute
Manian	Prasad	M.D.	Texas Heart Institute
Sohail	M. Rizwan	M.D.	Texas Heart Institute
Postalian	Alexander	M.D.	Texas Heart Institute
Hinsu	Punit	PharmD.	Texas Heart Institute
Watson	Carolyn		Texas Heart Institute
Chen	James	RN	Texas Heart Institute
Fink	Melyssa		Texas Heart Institute
Sturgis	Lydia		Texas Heart Institute
Walker	Kim		Texas Heart Institute
Mahon	Kim		Texas Heart Institute
Parenti	Jennifer	RN	Texas Heart Institute
Kappenman	Casey	MS	Texas Heart Institute
Knight	Aryn		Texas Heart Institute
Sturek	Jeffrey M.	M.D., Ph.D.	University of Virginia Health Systems
Barros	Andrew	M.D., M.S.	University of Virginia Health Systems
Enfield	Kyle B.	M.D., F.C.C.M., S.H.E.A.	University of Virginia Health Systems
Kadl	Alexandra	M.D.	University of Virginia Health Systems
Green	China J.	B.S., C.C.R.C.	University of Virginia Health Systems
Simon	Rachel M.	R.N., B.S.N., C.C.R.C.	University of Virginia Health Systems
Fox	Ashley	B.S.	University of Virginia Health Systems
Thornton	Kara	PharmD., M.Ed., C.C.R.P.	University of Virginia Health Systems
Adams	Amy	PharmD., C.C.R.P.	University of Virginia Health Systems
Badhwar	Vinay	M.D.	West Virginia University
Sharma	Sunil	M.D.	West Virginia University
Peppers	Briana	D.O.	West Virginia University
McCarthy	Paul	M.D.	West Virginia University
Krupica	Troy	M.D.	West Virginia University
Sarwari	Arif	M.D., M.S., M.B.A.	West Virginia University
Reece	Rebecca	M.D.	West Virginia University
Fornaresico	Lisa	Ph.D.	West Virginia University
Glaze	Chad	M.S.	West Virginia University
Evans	Raquel	B.S.N., R.N.	West Virginia University

Di	Fang	R.N., M.S.N.	West Virginia University
Carlson	Shawn	M.D., M.S.	West Virginia University
Aucremanne	Tanja	B.S.N., R.N.	West Virginia University
Tennant	Connie	B.S.N., R.N.	West Virginia University
Giblin Sutton	Lisa	Pharm.D.	West Virginia University
Buterbaugh	Sabrina	Pharm.D.	West Virginia University
Williams	Roger	C.Ph.T.	West Virginia University
Bunner	Robin	B.S.	West Virginia University
Traverse	Jay H.	M.D.	Minneapolis Heart Institute Foundation
Rhame	Frank	M.D.	Minneapolis Heart Institute Foundation
Huelster	Joshua	M.D.	Minneapolis Heart Institute Foundation
Kethireddy	Rajesh	M.D.	Minneapolis Heart Institute Foundation
Davies	Irena	CCRC	Minneapolis Heart Institute Foundation
Salamanca	Julianne	MS	Minneapolis Heart Institute Foundation
Majeski	Christine	RN, CCRC	Minneapolis Heart Institute Foundation
Skelton	Paige	PharmD.	Minneapolis Heart Institute Foundation
Zarambo	Maria	PharmD., B.C.O.P	Minneapolis Heart Institute Foundation
Sarafolean	Andrea	RN, CCRC	Minneapolis Heart Institute Foundation
Bowdish	Michael E.	M.D., M.S.	University of Souther California
Borok	Zea	M.B., Ch.B.	University of Souther California
Wald-Dickler	Noah	M.D.	University of Souther California
Hutcheon	Douglass	M.D.	University of Souther California
Towfighi	Amytis	M.D.	University of Souther California
Lee	Mary	M.D.	University of Souther California
Lewis	Meghan R.	M.D.	University of Souther California
Spellberg	Brad	M.D.	University of Souther California
Sher	Linda	M.D.	University of Souther California
Sharma	Aniket	M.D.	University of Souther California
Olds	Anna P.	M.D.	University of Souther California
Justino	Chris	P.A.-C.	University of Souther California
Loxano	Edward	M.D.	University of Souther California
Romero	Chris	C.R.C.	University of Souther California
Leong	Janet	C.R.C.	University of Souther California
Rodina	Valentina	M.D.	University of Souther California
Quesada	Christine	C.R.C.	University of Souther California
Hamilton	Luke		University of Souther California
Escobar	Jose		University of Souther California
Leshnower	Brad	M.D., F.A.C.S.	Emory University
Bender	William	M.D., M.P.H.	Emory University
Sharifpour	Milad	M.D., M.S.	Emory University
Miller	Jeffrey	M.D.	Emory University
Farrington	Woodrow	M.D.	Emory University
Baio	Kim T.	R.N., M.S.N.	Emory University
McBride	Mary	R.N., B.S.N., M.A.S.	Emory University
Fielding	Michele	R.N., B.S.N., C.C.R.C.	Emory University
Mathewson	Sonya	R.N., B.S.N., C.C.R.C.	Emory University
Porte	Kristina	B.A:, C.C.R.C.	Emory University

Maton	Missy	R.N., B.S.N.	Emory University
Ponder	Chari	R.N., B.S.N.	Emory University
Haley	Elisabeth	R.N., B.S.N., C.C.R.C.	Emory University
Spainhour	Christine	R.N., C.C.R.C.	Emory University
Rogers	Susan	R.Ph.	Emory University
Tyler	Derrick	C.C.R.P.	Emory University
Madathil	Ronson J.	M.D.	University of Maryland
Rabin	Joseph	M.D.	University of Maryland
Levine	Andrea	M.D.	University of Maryland
Saharia	Kapil	M.D.	University of Maryland
Tabatabai	Ali	M.D.	University of Maryland
Lau	Christine	M.D., M.B.A.	University of Maryland
Gammie	James S.	M.D.	University of Maryland
Peguero	Maya-Loren		University of Maryland
McKernan	Kimberly		University of Maryland
Audette	Mathew		University of Maryland
Fleischmann	Emily		University of Maryland
Akbari	Kreshta	M.S.	University of Maryland
Lee	Myounghee	Ph.D., Pharm. D.	University of Maryland
Chi	Andrew	Pharm.D.	University of Maryland
Salehi	Hanna	Pharm.D.	University of Maryland
Pariser	Alan	Pharm.D.	University of Maryland
Nyguyen	Phuong Tran	Pharm.D.	University of Maryland
Moore	Jessica		University of Maryland
Gee	Adrienne		University of Maryland
Vincent	Shelika		University of Maryland
Zuckerman	Richard A.	M.D., M.P.H.	Dartmouth-Hitchcock Medical Center
Iribarne	Alexander	M.D., M.S.	Dartmouth-Hitchcock Medical Center
Metzler	Sara	B.S.N., R.N.	Dartmouth-Hitchcock Medical Center
Shipman	Samantha	B.S.N., R.N.	Dartmouth-Hitchcock Medical Center
Johnson	Haley		Dartmouth-Hitchcock Medical Center
Newton	Crystallee	B.A., C.C.R.C.	Dartmouth-Hitchcock Medical Center
Parr	Doug	Pharm.D.	Dartmouth-Hitchcock Medical Center
Miller	Leslie	M.D.	BayCare Health System
Schelle	Beth	R.N.	BayCare Health System
McLean	Sherry	R.N.	BayCare Health System
Rothbaum	Howard R.	M.D.	BayCare Health System
Alvarez	Michael S.	D.O.	BayCare Health System
Kalan	Shivam P.	M.D.	BayCare Health System
Germann	Heather H.	M.D.	BayCare Health System
Hendershot	Jennifer	Pharm.D., B.C.C.C.P.	BayCare Health System
Moroney	Karen	R.N.	BayCare Health System
Herring	Karen	R.N.	BayCare Health System
Cook	Sharri	R.R.T.	BayCare Health System
Paul	Pam		BayCare Health System
Walker-Ignasiak	Rebecca		BayCare Health System

**PETAL**	North	Crystal	M.D.	Prevention and Early Treatment of Acute Lung Injury (PETAL) ICC, Massachusetts General Hospital, Boston, Massachusetts, USA
	Oldmixon	Cathryn	R.N.	Prevention and Early Treatment of Acute Lung Injury (PETAL) ICC, Massachusetts General Hospital, Boston, Massachusetts, USA
	Ringwood	Nancy	B.S.N.	Prevention and Early Treatment of Acute Lung Injury (PETAL) ICC, Massachusetts General Hospital, Boston, Massachusetts, USA
	Muzikansky	Ariela	R.N., B.A./B.S.	Prevention and Early Treatment of Acute Lung Injury (PETAL) ICC, Massachusetts General Hospital, Boston, Massachusetts, USA
	Morse	Richard	B.A./B.S.	Prevention and Early Treatment of Acute Lung Injury (PETAL) ICC, Massachusetts General Hospital, Boston, Massachusetts, USA
	Fitzgerald	Laura	B.A./B.S.	Prevention and Early Treatment of Acute Lung Injury (PETAL) ICC, Massachusetts General Hospital, Boston, Massachusetts, USA
	Morin	Haley D.	B.S.N.	Prevention and Early Treatment of Acute Lung Injury (PETAL) ICC, Massachusetts General Hospital, Boston, Massachusetts, USA
	Brower	Roy G.	M.D.	PETAL Steering Committee Chair, Johns Hopkins University
	Reineck	Lora A.	M.D., M.S.	U.S. National Heart Lung and Blood Institute
	Bienstock,	Karen	PA-C, M.S.	U.S. National Heart Lung and Blood Institute
	Steingrub	Jay H.	M.D.	ALIGNE Site Coordinating Center (SCC) Lead Investigators, Baystate Medical Center
	Hou	Peter K.	M.D.	ALIGNE Site Coordinating Center (SCC) Lead Investigators, Brigham and Women's Hospital
	Steingrub	Jay S.	M.D.	Baystate Medical Center
	Tidswell	Mark A.	M.D.	Baystate Medical Center
	Kozikowski	Lori-Ann	R.N., B.S.N., C.C.R.N.	Baystate Medical Center
	Kardos	Cynthia	R.N., B.S.N., C.C.R.N.	Baystate Medical Center
	DeSouza	Leslie		Baystate Medical Center
	Romain	Sarah	R.N., B.S.N.	Baystate Medical Center
	Thornton-Thompson	Sherell		Baystate Medical Center
	Talmor	Daniel	M.D.	Boston SCC Lead Investigators, Beth Israel Deaconess Medical Center
	Shapiro	Nathan	M.D.	Boston SCC Lead Investigators, Beth Israel Deaconess Medical Center
	Andromidas,	Konstantinos		Beth Israel Deaconess Medical Center
	Banner-Goodspeed,	Valerie	M.P.H.	Beth Israel Deaconess Medical Center
	Bolstad	Michael		Beth Israel Deaconess Medical Center
	Boyle,	Katherine L.	M.D.	Beth Israel Deaconess Medical Center
	Cabrera	Payton		Beth Israel Deaconess Medical Center
	deVilla,	Arnaldo	R.N., M.P.H.	Beth Israel Deaconess Medical Center
	Ellis,	Joshua C.	M.D.	Beth Israel Deaconess Medical Center
	Grafals,	Ana		Beth Israel Deaconess Medical Center
	Hayes	Sharon	R.N.	Beth Israel Deaconess Medical Center
	Higgins	Conor		Beth Israel Deaconess Medical Center
	Kurt	Lisa		Beth Israel Deaconess Medical Center
	Kurtzman,	Nicholas	M.D.	Beth Israel Deaconess Medical Center
	Redman,	Kimberly	R.N., B.S.N.	Beth Israel Deaconess Medical Center
	Rosseto	Elinita		Beth Israel Deaconess Medical Center
	Scaffidi	Douglas		Beth Israel Deaconess Medical Center

Shapiro,	Nathan	M.D., M.P.H.	Beth Israel Deaconess Medical Center
Filbin,	Michael R.	M.D., M.Sc.	Massachusetts General Hospital
Hibbert,	Kathryn A.	M.D.	Massachusetts General Hospital
Parry,	Blair	C.C.R.C., B.A.	Massachusetts General Hospital
Margolin,	Justin	B.S.	Massachusetts General Hospital
Hillis,	Brooklynn	B.S.N, R.N.	Massachusetts General Hospital
Hamer	Rhonda		Massachusetts General Hospital
Brait	Kelsey	B.B.A., B.Sc.	Massachusetts General Hospital
Beakes	Caroline	B.S.	Massachusetts General Hospital
McKaig	Brenna	B.S.	Massachusetts General Hospital
Kugener	Eleonore	B.A.	Massachusetts General Hospital
Jones	Alan E.	M.D.	University of Mississippi
Galbraith	James	M.D.	University of Mississippi
Nandi	Utsav	M.D.	University of Mississippi
Peacock	Rebekah	R.N.	University of Mississippi
Hendey	Gregory	M.D.	California SCC Lead Investigators, David Geffen School of Medicine at UCLA
Kangelaris	Kirsten	M.D., M.A.S.	University of California San Francisco
Ashktorab	Kimia	B.A.	University of California San Francisco
Gropper	Rachel	B.S.	University of California San Francisco
Agrawal	Anika	B.S.	University of California San Francisco
Yee	Kimberley J.	B.Sc.	University of California San Francisco
Jauregui	Alejandra E.	B.A.	University of California San Francisco
Zhuo	Hanjing	M.P.H.	University of California San Francisco
Almasri	Eyad	M.D.	University of California Fresno
Fayed	Mohamed	M.D.	University of California Fresno
Hubel	Kinsley A.	M.D.	University of California Fresno
Hughes	Alyssa R.	B.S.	University of California Fresno
Garcia	Rebekah L.	C.C.R.P.	University of California Fresno
Lim	George W.	M.D.	Ronald Reagan UCLA Medical Center
Chang	Steven Y.	M.D.	Ronald Reagan UCLA Medical Center
Hendey	Gregory	M.D.	Ronald Reagan UCLA Medical Center
Lin	Michael Y.	M.D.	Ronald Reagan UCLA Medical Center
Vargas	Julia	B.S.	Ronald Reagan UCLA Medical Center
Sihota	Hena	B.S.	Ronald Reagan UCLA Medical Center
Beutler	Rebecca	M.S.	Ronald Reagan UCLA Medical Center
Agarwal	Trisha		Ronald Reagan UCLA Medical Center
Wilson,	Jennifer G.	M.D., M.S.	Stanford University
Vojnik,	Rosemary	B.S.	Stanford University
Perez,	Cynthia	B.S.	Stanford University
McDowell,	Jordan H.	M.S.	Stanford University
Roque	Jonasel	B.S.	Stanford University
Wang	Henry	M.D., M.S.	University of Texas Health Science Center
Huebinger	Ryan M.	M.D.	University of Texas Health Science Center
Patel	Bela	M.D.	University of Texas Health Science Center
Vidales	Elizabeth	M.P.H., B.M.S.	University of Texas Health Science Center
Albertson	Timothy	M.D.	University of California Davis Health
Hardy	Erin	B.A./B.S.	University of California Davis Health
Harper	Richart	M.D.	University of California Davis Health

Moss	Marc A.	M.D.	Colorado SCC Lead Investigators, University of Colorado Hospital
Baduashvili	Amiran	M.D.	University of Colorado Hospital
Chauhan	Lakshmi	M.D.	University of Colorado Hospital
Douin	David J.	M.D.	University of Colorado Hospital
Martinez	Flora	R.N.	University of Colorado Hospital
Finck	Lani L.	M.P.H.	University of Colorado Hospital
Bastman	Jill	R.N.	University of Colorado Hospital
Howell	Michelle	R.N.	University of Colorado Hospital
Higgins	Carrie	R.N.	University of Colorado Hospital
McKeehan	Jeffrey	M.Sc.N.	University of Colorado Hospital
Finigan	Jay	M.D.	National Jewish Health/ St. Joseph Hospital
Stubenrauch	Peter	M.D.	National Jewish Health/ St. Joseph Hospital
Janssen	William J.	M.D.	National Jewish Health/ St. Joseph Hospital
Griesmer	Christine	R.N., M.P.H.	National Jewish Health/ St. Joseph Hospital
VerBurg	Olivia	B.A.	National Jewish Health/ St. Joseph Hospital
Hyzy	Robert C.	M.D.	Michigan SCC Lead Investigators, University of Michigan
Park	Pauline K.	M.D.	Michigan SCC Lead Investigators, University of Michigan
Nelson,	Kristine	R.N.	University of Michigan
McSparron,	Jake I.	M.D.	University of Michigan
Co,	Ivan N.	M.D.	University of Michigan
Wang,	Bonnie R.	M.D.	University of Michigan
Jimenez,	Jose	M.D.	University of Michigan
Olbrich	Norman		University of Michigan
McDonough	Kelli		University of Michigan
Jia	Shijing	M.D.	University of Michigan
Hanna	Sinan		University of Michigan
Gong	Michelle N.	M.D., M.S.	Montefiore-Sinai SCC Lead Investigators: Montefiore Medical Center
Richardson	Lynne D.	M.D.	Mount Sinai Hospital
Nair	Rahul	M.D.	Montefiore Medical Center Moses
Lopez	Brenda	M.D.	Montefiore Medical Center Moses
Amosu	Omowunmi	M.S.	Montefiore Medical Center Moses
Offor	Obiageli	M.D.	Montefiore Medical Center Moses
Tzehaie	Hiwet	B.S.	Montefiore Medical Center Moses
Nkemdirim	William	M.D.	Montefiore Medical Center Moses
Boujid	Sabah	B.S.	Montefiore Medical Center Moses
Mosier	Jarrod M.	M.D.	Banner University Medical Center Tucson
Hypes	Cameron	M.D.	Banner University Medical Center Tucson
Campbell	Elizabeth Salvagio	Ph.D.	Banner University Medical Center Tucson
Bixby	Billie	M.D.	Banner University Medical Center Tucson
Gilson	Boris	B.A./B.S.	Banner University Medical Center Tucson
Lopez	Anitza	B.S.	Banner University Medical Center Tucson
Bime	Christian	M.D.	Banner University Medical Center Tucson
Parthasarathy	Sairam	M.D.	Banner University Medical Center Tucson
Cano	Ariana M.	B.A., B.S.	Banner University Medical Center Tucson
Hite	R. Duncan	M.D.	Ohio SCC Lead Investigators, University of Cincinnati
Terndrup	Thomas E.	M.D.	Ohio State University
Wiedemann	Herbert P.	M.D., M.B.A.	Cleveland Clinic Foundation
Hudock	Kristin	M.D.	University of Cincinnati
Tanzeem	Hammad	M.D.	University of Cincinnati
More	Harshada	M.D.	University of Cincinnati

Martinkovic	Jamie	C.N.P.	University of Cincinnati
Sellers	Susan	R.N., B.S.N., C.C.R.P.	University of Cincinnati
Houston	Judy	PharmD.	University of Cincinnati
Burns	Mary	PharmD.	University of Cincinnati
Kiran	Simra	M.D.	University of Cincinnati
Roads	Tammy	C.C.R.P.	University of Cincinnati
Kennedy	Sarah	C.N.P.	University of Cincinnati
Duggal	Abhijit	M.D.	Cleveland Clinic Foundation, Cleveland Clinic Fairview Hospital
Thiruchelvam	Nirosshan	M.D.	Cleveland Clinic Foundation, Cleveland Clinic Fairview Hospital
Ashok	Kiran	B.S.	Cleveland Clinic Foundation, Cleveland Clinic Fairview Hospital
King	Alexander H.	M.S.	Cleveland Clinic Foundation, Cleveland Clinic Fairview Hospital
Mehkri	Omar	M.D.	Cleveland Clinic Foundation, Cleveland Clinic Fairview Hospital
Dugar	Siddharth	M.D.	Cleveland Clinic Foundation, Cleveland Clinic Fairview Hospital
Sahoo	Debasis	M.D.	Cleveland Clinic Foundation, Cleveland Clinic Fairview Hospital
Yealy	Donald M.	M.D.	University of Pittsburgh Medical Center
Angus	Derek C.	M.D.	University of Pittsburgh Medical Center
Weissman	Alexandra J.	M.D.	University of Pittsburgh Medical Center
Vita	Tina M.	R.N.	University of Pittsburgh Medical Center
Berryman	Emily	B.S., B.A.	University of Pittsburgh Medical Center
Hough	Catherine L.	M.D.	Pacific Northwest SCC Lead Investigators, Oregon Health and Science University
Khan	Akram	M.D.	Oregon Health and Science University
Krol	Olivia F.		Oregon Health and Science University
Mills	Emmanuel	M.D.	Oregon Health and Science University
Kinjal	Mistry		Oregon Health and Science University
Briceno	Genesis		Oregon Health and Science University
Reddy	Raju	M.D.	Oregon Health and Science University
Hubel	Kinsley	M.D.	Oregon Health and Science University
Jouzestani	Milad K.		Oregon Health and Science University
McDougal	Madeline	B.A., B.S.	Oregon Health and Science University
Deshmukh	Rupali		Oregon Health and Science University
Johnston	Nicholas J.	M.D.	Harborview Medical Center, University of Washington Medical Center
Robinson	Bryce H.	M.D.	Harborview Medical Center, University of Washington Medical Center
Gundel	Staphanie J.	R.D.	Harborview Medical Center, University of Washington Medical Center
Katsandres	Sarah C.	B.S.	Harborview Medical Center, University of Washington Medical Center
Chen	Peter	M.D.	Cedars-Sinai Medical Center
Torbati	Sam S.	M.D.	Cedars-Sinai Medical Center
Parimon	Tanyalak	M.D.	Cedars-Sinai Medical Center
Caudill	Antonina	M.P.H., C.P.H.	Cedars-Sinai Medical Center
Mattison	Brittany		Cedars-Sinai Medical Center
Jackman	Susan E.	B.S.N., M.S.	Cedars-Sinai Medical Center
Chen	Po-En	B.S.N.	Cedars-Sinai Medical Center
Bayoumi	Emad	M.D.	Cedars-Sinai Medical Center
Ojukwu	Cristabelle	B.S.	Cedars-Sinai Medical Center

Fine	Devin	B.S.	Cedars-Sinai Medical Center
Weissberg	Gwendolyn	B.S.	Cedars-Sinai Medical Center
Isip	Katherine	B.S.	Cedars-Sinai Medical Center
Choi-Kuaea	Yunhee	M.S.W.	Cedars-Sinai Medical Center
Mehdikhani	Shaunt	M.S.	Cedars-Sinai Medical Center
Dar	Tahir B.	Ph.D.	Cedars-Sinai Medical Center
Augustin	Nsole Biteghe Fleury	Ph.D.	Cedars-Sinai Medical Center
Tran	Dana	B.S.	Cedars-Sinai Medical Center
Dukov	Jennifer Emilow	B.S.	Cedars-Sinai Medical Center
Matusov	Yuri	M.D.	Cedars-Sinai Medical Center
Choe	June	M.D.	Cedars-Sinai Medical Center
Hindoyan	Niree A.	B.S.	Cedars-Sinai Medical Center
Wynter	Timothy	B.S.	Cedars-Sinai Medical Center
Pascual	Ethan	M.A.	Cedars-Sinai Medical Center
Clapham	Gregg J.	M.A:	Cedars-Sinai Medical Center
Herrera	Lisa		Cedars-Sinai Medical Center
Caudill	Antonia	M.P.H., C.P.H.	Cedars-Sinai Medical Center
O'Mahony	D. Shane	M.D.	Swedish Hospital First Hill
Nyatsatsang	Sonam T.	M.D.	Swedish Hospital First Hill
Wilson	David M.	M.D.	Swedish Hospital First Hill
Wallick	Julie A.	B.A./B.S.	Swedish Hospital First Hill
Duven	Alexandria M.	R.N.	Swedish Hospital First Hill
Fletcher	Dakota D.	B.S.	Swedish Hospital First Hill
Miller	Chadwick	M.D.	Wake Forest Baptist Health
Files	D. Clark	M.D.	Wake Forest Baptist Health
Gibbs	Kevin W.	M.D.	Wake Forest Baptist Health
Flores,	Lori S.	D.N.P.	Wake Forest Baptist Health
LaRose	Mary E.	R.N., B.S.N.	Wake Forest Baptist Health
Landreth	Leigha D.	R.N., B.S.N.	Wake Forest Baptist Health
Palacios	D. Rafael	B.S.C.R.	Wake Forest Baptist Health
Parks	Lisa	R.N.	Wake Forest Baptist Health
Hicks	Madeline	B.A.	Wake Forest Baptist Health
Goodwin	Andrew J.	M.D.	Medical Center of South Carolina
Kilb	Edward F.	M.D.	Medical Center of South Carolina
Lematty	Caitlan T.	B.S.	Medical Center of South Carolina
Patti	Kerilyn		Medical Center of South Carolina
Grady	Abigail	B.S.	Medical Center of South Carolina
Rasberry	April	B.S.	Medical Center of South Carolina
Morris	Peter E.	M.D.	University of Kentucky
Sturgill	Jamie L.	Ph.D.	University of Kentucky
Cassity	Evan P.	M.S.	University of Kentucky
Dhar	Sanjay	M.D.	University of Kentucky
Montgomery-Yates	Ashley A.	M.D.	University of Kentucky
Pasha	Sarah N.	M.D.	University of Kentucky
Mayer	Kirby P.	Ph.D.	University of Kentucky
Bissel	Brittany	Pharm.D., Ph.D.	University of Kentucky
Trott	Terren	M.D.	University of Kentucky
Rehman	Shahnaz	M.D.	University of Kentucky

	de Wit	Marjolein	M.D.	Virginia Commonwealth University
	Mason	Jessica	M.P.H.	Virginia Commonwealth University
	Bledsoe	Joseph	M.D.	Intermountain Medical Center
	Knowlton	Kirk U.	M.D.	Intermountain Medical Center
	Brown	Samuel	M.D.	Intermountain Medical Center
	Lanspa	Michael	M.D.	Intermountain Medical Center
	Leither	Lindsey	M.D.	Intermountain Medical Center
	Pelton	Ithan	M.D.	Intermountain Medical Center
	Armbruster	Brent P.	B.S.	Intermountain Medical Center
	Montgomery	Quinn	B.S., A.E.M.T.	Intermountain Medical Center
	Kumar	Naresh	M.P.H., C.C.R.P.	Intermountain Medical Center
	Fergus	Melissa	B.S.	Intermountain Medical Center
	Imel	Karah	A.S., C.C.R.P.	Intermountain Medical Center
	Palmer	Ghazal	PharmD.	Intermountain Medical Center
	Webb	Brandon	M.D.	Intermountain Medical Center
	Klippel	Carolyn	B.S.	Intermountain Medical Center
	Jensen	Hannah	B.S.	Intermountain Medical Center
	Duckworth	Sarah		Intermountain Medical Center
	Gray	Andrew	B.S.	Intermountain Medical Center
	Burke	Tyler	B.S.	Intermountain Medical Center
	Knox	Dan	M.D.	Intermountain Medical Center
	Lumpkin	Jenna	B.S.	Intermountain Medical Center
	Aston	Valerie T.	M.B.A., R.R.T., C.C.R.P.	Intermountain Medical Center
	Applegate	Darrin	B.S.	Intermountain Medical Center
	Serezlic	Erna	B.S.	Intermountain Medical Center
	Brown	Katie	B.S., R.N.	Intermountain Medical Center
	Merril	Mardee	B.S., C.C.R.P.	Intermountain Medical Center
	Harris	Estelle S.	M.D.	University of Utah
	Middleton	Elizabeth A.	M.D.	University of Utah
	Barrios	Macy A.G.	B.S.	University of Utah
	Greer	Jorden	B.S.	University of Utah
	Schmidt	Amber D.	B.S	University of Utah
	Webb	Melissa K.	Pharm.D.	University of Utah
	Paine	Roert	M.D.	University of Utah
	Callahan	Sean J.	M.D.	University of Utah
	Waddoups	Lindsey J.	M.S.	University of Utah
	Yamane	Misty B.	B.S.	University of Utah
	Self	Wesley H.	M.D., M.P.H.	Vanderbilt SCC Lead Investigators, Vanderbilt University Medical Center
	Rice	Todd W.	M.D., M.S.C.I.	Vanderbilt SCC Lead Investigators, Vanderbilt University Medical Center
	Casey	Jonathan D.	M.D., M.S.C.I.	Vanderbilt University Medical Center
	Johnson	Jakea	M.P.H.	Vanderbilt University Medical Center
	Gray	Christopher	R.N.	Vanderbilt University Medical Center
	Hays	Margaret	R.N.	Vanderbilt University Medical Center
	Roth	Megan	R.N.	Vanderbilt University Medical Center
				Divison of Clinical Research, NIAID, NIH, ICC
**DCR**	Menon	Vidya	M.D, F.A.C.P.	Lincoln Medical Center

Kasubhai	Moiz	M.D.	Lincoln Medical Center
Pillai	Anjana	M.D.	Lincoln Medical Center
Daniel	Jean	M.D., M.A.C.P.	Lincoln Medical Center
Sittler	Daniel	M.D.	Lincoln Medical Center
Kanna	Balavenkatesh	M.D., M.P.H., F.A.C.P.	Lincoln Medical Center
Jilani	Nargis	M.D.	Lincoln Medical Center
Amaro	Francisco	R.N., F.N.P.,-BC.	Lincoln Medical Center
Santana	Jessica	B.A.	Lincoln Medical Center
Lyakovestsky	Aleksandr	PharmD., B.C.P.S.	Lincoln Medical Center
Madhoun	Issa	PharmD.	Lincoln Medical Center
Desroches	Louis Marie	R.P.H.	Lincoln Medical Center
Amadon	Nicole	PharmD., B.C.G.P.	Lincoln Medical Center
Bahr	Alaa	PharmD., B.C.P.S.	Lincoln Medical Center
Ezzat	Imaan	PharmD., B.H.S.A., M.A.	Lincoln Medical Center
Guerrero	Maryanne		Lincoln Medical Center
Padilla	Joane		Lincoln Medical Center
Fullmer	Jessie		Lincoln Medical Center
Singh	Inderpreet		Lincoln Medical Center
Shah	Syed Hamad Ali		Lincoln Medical Center
Narang	Rajeev	M.D.	CHRISTUS Spohn Shoreline Hospital
Mock	Polly	R.N., C.C.R.C., C.H.R.C.	CHRISTUS Spohn Shoreline Hospital
Shadle	Melissa	R.N., B.S.N., O.C.N., C.C.R.C.	CHRISTUS Spohn Shoreline Hospital
Hernandez	Brenda	R.N.	CHRISTUS Spohn Shoreline Hospital
Welch	Kevin	PharmD.	CHRISTUS Spohn Shoreline Hospital
Payne	Andrea	PharmD.	CHRISTUS Spohn Shoreline Hospital
Ertl	Gabriela	PharmD.	CHRISTUS Spohn Shoreline Hospital
Canario	Daniel	M.D.	Hendrick Medical Center
Barrientos	Isabel	M.S.N., A.R.P.N.	Hendrick Medical Center
Goss	Danielle	M.P.H., M.H.A.	Hendrick Medical Center
DeVries	Mattie	RPh., PharmD.	Hendrick Medical Center
Folowosele	Ibidolapo	RPh.	Hendrick Medical Center
Garner	Dorothy	M.D.	Carilion Roanoke Memorial Hospital
Gomez	Mariana	M.D.	Carilion Roanoke Memorial Hospital
Price	Justin	M.D.	Carilion Roanoke Memorial Hospital
Bansal	Ekta	M.D.	Carilion Roanoke Memorial Hospital
Wong	Jim	M.D.	Carilion Roanoke Memorial Hospital
Faulhaber	Jason	M.D.	Carilion Roanoke Memorial Hospital
Fazili	Tasaduq	M.D.	Carilion Roanoke Memorial Hospital
Yeary	Brian	M.D.	Carilion Roanoke Memorial Hospital
Ndolo	Ruth	R.N.	Carilion Roanoke Memorial Hospital

	Bryant	Christina	R.N.	Carilion Roanoke Memorial Hospital
	Smigeil	Bridgette	PharmD.	Carilion Roanoke Memorial Hospital
	Robinson	Philip	M.D.	Hoag Memorial Hospital Presbyterian
	Najjar	Rana	M.S.H.C.A., C.R.C.	Hoag Memorial Hospital Presbyterian
	Jones	Patrice	C.R.C.	Hoag Memorial Hospital Presbyterian
	Nguyen	Julie	R.R.T., C.R.C.	Hoag Memorial Hospital Presbyterian
	Chin	Christina	PharmD.	Hoag Memorial Hospital Presbyterian
	Taha	Hassan	M.D.	Cotton O’Neil Clinical Research Center
	Najm	Salah	M.D., M.B.A.	Cotton O’Neil Clinical Research Center
	Smith	Christopher	PharmD.	Cotton O’Neil Clinical Research Center
	Moore	Jason	PharmD.	Cotton O’Neil Clinical Research Center
	Nassar	Talal	PharmD.	Cotton O’Neil Clinical Research Center
	Gallinger	Nick	PharmD.	Cotton O’Neil Clinical Research Center
	Christian	Amy	R.N., C.C.R.C.	Cotton O’Neil Clinical Research Center
	Mauer	D’Amber	R.N., B.S.N.	Cotton O’Neil Clinical Research Center
	Phipps	Ashley	R.N., B.S.N.	Cotton O’Neil Clinical Research Center
	Waters,	Michael	M.D.	Velocity Chula Vista
	Zepeda	Karla	N.P.	Velocity Chula Vista
	Coslet	Jordan	P.A.	Velocity Chula Vista
	Landazuri	Rosalynn	B.S., C.R.C.	Velocity Chula Vista
	Pineda	Jacob	C.R.C.	Velocity Chula Vista
	Uribe	Nicole	RPh.	Velocity Chula Vista
	Garcia	Jose Ruiz	CPhT.	Velocity Chula Vista
	Barbabosa	Cecilia	R.N.	Velocity Chula Vista
	Sandler	Kaitlyn	B.S.N.	Velocity Chula Vista
	Overcash	J. Scott	M.D.	Velocity San Diego
	Marquez	Adrienna		Velocity San Diego
	Chu	Hanh	M.S.N., NP-C.	Velocity San Diego
	Lee	Kia	M.S.N., A.N.P.-B.C.	Velocity San Diego
	Quillin	Kimberly	B.S.N., R.N.	Velocity San Diego
	Garcia	Andrea	M.S.N., R.N.	Velocity San Diego
	Lew	Pauline	PharmD.	Velocity San Diego
	Rogers	Ralph	M.D.	Rhode Island Hospital; The Miriam Hospital
	Shehadeh	Fadi	M.Sc.	Rhode Island Hospital; The Miriam Hospital
	Mylona	Evangelia K.	M.Sc.	Rhode Island Hospital; The Miriam Hospital
	Kaczynski	Matthew	B.Sc.	Rhode Island Hospital; The Miriam Hospital
	Tran	Quynh-Lam	B.Sc.	Rhode Island Hospital; The Miriam Hospital
	Benitez	Gregorio	M.P.H.	Rhode Island Hospital; The Miriam Hospital
	Mishra	Biswajit	Ph.D.	Rhode Island Hospital; The Miriam Hospital
	Felix	Lewis Oscar	Ph.D.	Rhode Island Hospital; The Miriam Hospital
	Vafea	Maria Tsikala	M.D.	Rhode Island Hospital; The Miriam Hospital
	Atalla	Eleftheria	M.D.	Rhode Island Hospital; The Miriam Hospital
	Davies	Robin	B.S.N., B.A., R.N.	Rhode Island Hospital; The Miriam Hospital
	Hedili	Salma	C.P.T.	Rhode Island Hospital; The Miriam Hospital
	Monkeberg	Maria Andrea	M.S., R.Ph., B.C.O.P.	Rhode Island Hospital
	Tabler	Sandra	R.Ph., B.C.O.P.	Rhode Island Hospital

	Harrington	Britt	Pharm.D.	The Miriam Hospital
	Meegada	Sreenath	M.D.,	Christus Good Shepard
	Koripalli	VenkataSandeep	M.D.	Christus Good Shepard
	Muddana	Prithvi	M.D.	Christus Good Shepard
	Jain	Lakshay	M.D.	Christus Good Shepard
	Undavalli	Chaitanya	M.D.	Christus Good Shepard
	Kavya	Parasa	M.D.	Christus Good Shepard
	Ibiwoye	Mofoluwaso	M.D.	Christus Good Shepard
	Akilo	Hameed	M.D.	Christus Good Shepard
	Lovette,	Bryce D.	PharmD.	Christus Good Shepard
	Wylie,	Jamie-Crystal	MHA, FACHE,	Christus Good Shepard
	Smith,	Diana M.	BS	Christus Good Shepard
	Poon	Kenneth	M.D., F.A.C.P.	Memorial Health Care System
	Eckardt,	Paula	M.D., F.A.C.P., F.I.D.S.A., A.A.H.I.V.S.	Memorial Health Care System
	Heysu	Rubio-Gomez,	M.D., F.A.C.P., F.I.D.S.A.	Memorial Health Care System
	Sundararaman,	Nithya	M.A., M.S., M.B.A.	Memorial Health Care System
	Alaby,	Doris	B.S.N., R.N.	Memorial Health Care System
	Sareli	Candice	M.D.	Memorial Health Care System
**WDC ICC**	Sánchez	Adriana	M.S.	INSIGHT Washington ICC, Veterans Affairs (VA) Medical Center, Washington, DC
	Popielski	Laura	M.P.H.	INSIGHT Washington ICC, Veterans Affairs (VA) Medical Center, Washington, DC
	Kambo	Amy	M.P.H.	INSIGHT Washington ICC, Veterans Affairs (VA) Medical Center, Washington, DC
	Viens	Kimberley	B.S., C.C.R.P.	INSIGHT Washington ICC, Veterans Affairs (VA) Medical Center, Washington, DC
	Turner	Melissa	M.S.W.	INSIGHT Washington ICC, Veterans Affairs (VA) Medical Center, Washington, DC
	Vjecha	Michael J.	M.D.	INSIGHT Washington ICC, Veterans Affairs (VA) Medical Center, Washington, DC
	Weintrob	Amy	M.D.	INSIGHT Washington ICC, Veterans Affairs (VA) Medical Center, Washington, DC
	Brar	Indira	M.D.	Henry Ford Health System
	Markowitz	Norman	M.D.	Henry Ford Health System
	Pastor	Erika	R.N	Henry Ford Health System
	Corpuz	Roweena	R.N	Henry Ford Health System
	Alangaden	George	M.D.	Henry Ford Health System
	McKinnon	John	M.D.	Henry Ford Health System
	Ramesh	Mayur	M.D.	Henry Ford Health System
	Herc	Erica	M.D.	Henry Ford Health System
	Yared	Nicholas	M.D.	Henry Ford Health System
	Abreu Lanfranco	Odaliz	M.D.	Henry Ford Health System
	Rivers	Emanuel	M.D.	Henry Ford Health System
	Swiderek	Jennifer	M.D.	Henry Ford Health System
	Hodari Gupta	Ariella	M.D.	Henry Ford Health System
	Pabla	Pardeep	Pharm D	Henry Ford Health System
	Eliya	Sonia	Pharm D	Henry Ford Health System

Jazrawi	Jehan	RpH	Henry Ford Health System
Delor	Jeremy	Pharm D	Henry Ford Health System
Desai	Mona	Pharm D	Henry Ford Health System
Cook	Aaron		Henry Ford Health System
Kathrina Jaehne	Anja		Henry Ford Health System
Kaur Gill	Jasreen		Henry Ford Health System
Renaud	Sheri		Henry Ford Health System
Sarveswaran	Siva		Henry Ford Health System
Gardner	Edward	M.D.	Public Health Institute at Denver Health
Scott	James	RN	Public Health Institute at Denver Health
Bianchini	Monica	Pharm D	Public Health Institute at Denver Health
Melvin	Casey	Pharm D	Public Health Institute at Denver Health
Kim	Gina	Pharm D	Public Health Institute at Denver Health
Wyles	David	M.D.	Public Health Institute at Denver Health
Kamis	Kevin		Public Health Institute at Denver Health
Miller	Rachel		Public Health Institute at Denver Health
Douglas	Ivor	MD	Public Health Institute at Denver Health
Haukoos	Jason		Public Health Institute at Denver Health
Hicks	Carrie		Public Health Institute at Denver Health
Lazarte	Susana	MD	Parkland Health and Hospital Systems
Marines-Price	Rubria	Ph.D., D.N.P., A.P.R.N.	Parkland Health and Hospital Systems
Osuji	Alice	R.N., B.S.N., M.S.N.	Parkland Health and Hospital Systems; University of Texas Southwestern Medical Center
Agbor	Barbine Tchamba Agbor	M.D.	Parkland Health and Hospital Systems
Petersen	Tianna	M.Sc., M.S.N.	Parkland Health and Hospital Systems; University of Texas Southwestern Medical Center
Kamel	Dena	B.S.	Parkland Health and Hospital Systems; University of Texas Southwestern Medical Center
Hansen	Laura	M.A.	Parkland Health and Hospital Systems; University of Texas Southwestern Medical Center
Garcia	Angie	M.D.	Parkland Health and Hospital Systems; University of Texas Southwestern Medical Center
Cha	Christine	PharmD.	Parkland Health and Hospital Systems
Mozaffari	Azadeh	PharmD.	Parkland Health and Hospital Systems
Hernandez	Rosa	PharmD., M.B.A.	Parkland Health and Hospital Systems
Cutrell	James	MD	University of Texas Southwestern Medical Center
Agbor	Barbine Tchamba Agbor	M.D.	University of Texas Southwestern Medical Center
Kim	Mina	PharmD.	University of Texas Southwestern Medical Center
DellaValle	Natalie	PharmD., B.C.P.S.	University of Texas Southwestern Medical Center
Gonzales	Sonia	PharmD., B.C.O.P.	University of Texas Southwestern Medical Center
Somboonwit	Charurut	M.D.	University of South Florida, Tampa General Hospital
Oxner	Asa	M.D.	University of South Florida, Tampa General Hospital
Guerra	Lucy	M.D.	University of South Florida, Tampa General Hospital
Hayes	Michael	PharmD.	University of South Florida, Tampa General Hospital
Nguyen	Thi	PharmD.	University of South Florida, Tampa General Hospital
Tran	Thanh	M.P.H.	University of South Florida, Tampa General Hospital
Pinto	Avenette		University of South Florida, Tampa General Hospital

Hatlen	Timothy	M.D.	Lundquist Institute for Biomedical Innovation
Anderson	Betty	B.S.	Lundquist Institute for Biomedical Innovation
Zepeda-Gutierrez	Ana	B.S.	Lundquist Institute for Biomedical Innovation
Martin	Dannae	B.A.	Lundquist Institute for Biomedical Innovation
Temblador	Cindi		Lundquist Institute for Biomedical Innovation
Cuenca	Avon	B.A.	Lundquist Institute for Biomedical Innovation
Tanoviceanu	Roxanne	PharmD.	Lundquist Institute for Biomedical Innovation
Prieto	Martha	PharmD.	Lundquist Institute for Biomedical Innovation
Guerrero	Mario	M.D.	Lundquist Institute for Biomedical Innovation
Martin	Dannae		Lundquist Institute for Biomedical Innovation
Daar	Eric	M.D.	Lundquist Institute for Biomedical Innovation
Correa	Ramiro		Lundquist Institute for Biomedical Innovation
Hartnell	Gabe		Lundquist Institute for Biomedical Innovation
Wortmann	Glenn	M.D.	Medstar Health Research Institute
Doshi	Saumil	M.D.	Medstar Health Research Institute
Moriarty	Theresa	M.S.N.R.N.	Medstar Health Research Institute
Gonzales	Melissa	C.R.C., III	Medstar Health Research Institute
Garman	Kristin	C.R.N.	Medstar Health Research Institute
Baker	Jason V.	M.D.	Hennepin Healthcare Research Institute
Frosch	Anne	M.D.	Hennepin Healthcare Research Institute
Goldsmith	Rachael	B.Sc.	Hennepin Healthcare Research Institute
Driver	Brian	M.D.	Hennepin Healthcare Research Institute
Frank	Christine	PharmD	Hennepin Healthcare Research Institute
Leviton	Tzivia	PharmD	Hennepin Healthcare Research Institute
Prekker	Matthew	M.D.	Hennepin Healthcare Research Institute
Jibrell	Hodan	B.Sc.	Hennepin Healthcare Research Institute
Lo	Melanie	M.D.	Hennepin Healthcare Research Institute
Klaphake	Jonathan	B.Sc.	Hennepin Healthcare Research Institute
Mackedanz	Shari	R.N.	Hennepin Healthcare Research Institute
Ngo	Linh	M.D.	Hennepin Healthcare Research Institute
Garcia-Myers	Kelly	B.Sc.	Hennepin Healthcare Research Institute
Kunisaki	Ken M.	M.D., M.S.	Minneapolis VA Medical Center
Wendt	Chris	M.D.	Minneapolis VA Medical Center
Melzer	Anne	M.D.	Minneapolis VA Medical Center
Wetherbee	Erin	M.D.	Minneapolis VA Medical Center
Drekonja	Dimitri	M.D.	Minneapolis VA Medical Center
Pragman	Alexa	M.D.	Minneapolis VA Medical Center
Hamel	Aimee	RN	Minneapolis VA Medical Center
Thielen	Abbie	PharmD.	Minneapolis VA Medical Center
Kunisaki	Ken M.	M.D., M.S.	Minneapolis VA Medical Center
Hassler	Miranda	B.A.	Minneapolis VA Medical Center
Walquist	Mary	B.S.	Minneapolis VA Medical Center
Augenbraun	Michael	M.D.	SUNY Downstate Medical Center
George	Jensen		SUNY Downstate Medical Center
Demeo	Lynette		SUNY Downstate Medical Center
Mishko	Motria	PharmD.	SUNY Downstate Medical Center
Thomas	Lorraine		SUNY Downstate Medical Center
Tatem	Luis		SUNY Downstate Medical Center
Dehovitz	Jack	M.D.	SUNY Downstate Medical Center
Abassi	Mahsa	D.O.	University of Minnesota
Leuck	Anne-Marie	M.D.	University of Minnesota
Rao	Via	M.S.	University of Minnesota

	Pullen	Matthew	M.D.	University of Minnesota
	Luke	Darlette	RPh	University of Minnesota
	LaBar	Derek	PharmD, BCPS	University of Minnesota
	Christiansen	Theresa	RPh	University of Minnesota
	Howard	Diondra		University of Minnesota
**VA ICC**	Biswas	Kousick	Ph.D.	INSIGHT US Department of Veterans Affairs (VA) ICC
	Harrington	Cristin	B.A.	INSIGHT US Department of Veterans Affairs (VA) ICC
	Garcia	Amanda	M.P.H.	INSIGHT US Department of Veterans Affairs (VA) ICC
	Bremer	Tammy		INSIGHT US Department of Veterans Affairs (VA) ICC
	Burke	Tara		INSIGHT US Department of Veterans Affairs (VA) ICC
	Koker	Brittany	B.S.	INSIGHT US Department of Veterans Affairs (VA) ICC
	Davis-Karim	Anne	PharmD.	INSIGHT US Department of Veterans Affairs (VA) ICC
	Pittman	David	B.E.	INSIGHT US Department of Veterans Affairs (VA) ICC
	Vasudeva	Shikha S.	M.D.	INSIGHT US Department of Veterans Affairs (VA) ICC
	Johnstone	Jaylynn R.	MPH	INSIGHT US Department of Veterans Affairs (VA) ICC
	Agnetti	Kate	B.S.	INSIGHT US Department of Veterans Affairs (VA) ICC
	Davis	Ruby	B.S.	INSIGHT US Department of Veterans Affairs (VA) ICC
	Trautner	Barbara	M.D., Ph.D.	Michael E. DeBakey VA Medical Center,
	Hines-Munson	Casey	B.S.	Michael E. DeBakey VA Medical Center,
	Van	John	B.A.	Michael E. DeBakey VA Medical Center,
	Dillon	Laura	M.Sc.	Michael E. DeBakey VA Medical Center,
	Wang	Yiqun	B.S., M.A.	Michael E. DeBakey VA Medical Center,
	Nagy-Agren	Stephanie	M.D.	Salem VA Medical Center
	Vasudeva	Shikha	M.D.	Salem VA Medical Center
	Ochalek	Tracy	B.S.N.	Salem VA Medical Center
	Caldwell	Erin	D.O.	Salem VA Medical Center
	Humerickhouse	Edward	M.D.	Salem VA Medical Center
	Boone	David	D.O.	Salem VA Medical Center
	McGraw	William	PharmD.	Salem VA Medical Center
	Looney	David J.	M.D.	VA San Diego Healthcare System
	Mehta	Sanjay R.	M.D.	VA San Diego Healthcare System
	Johns	Scott Thompson	PharmD.	VA San Diego Healthcare System
	St. John	Melissa		VA San Diego Healthcare System
	Raceles	Jacqueline	C.C.R.C.	VA San Diego Healthcare System
	Sear	Emily	B.S.N, R.N.	VA San Diego Healthcare System
	Funk	Stephen	PharmD.	VA San Diego Healthcare System
	Cesarini	Rosa		VA San Diego Healthcare System
	Fang	Michelle	PharmD.	VA San Diego Healthcare System
	Nicalo	Keith	R.N.	VA San Diego Healthcare System
	Drake	Wonder	M.D.	VA TVHS Nashville Campus
	Jones	Beatrice	M.S.N., R.N.	VA TVHS Nashville Campus
	Holtman	Teresa	DPh.	VA TVHS Nashville Campus
	Nguyen	Hien H.	M.D.	Sacramento VA Medical Center
	Maniar	Archana	M.D.	Sacramento VA Medical Center
	Johnson	Eric A.	M.D.	Sacramento VA Medical Center
	Nguyen	Lam	B.A.	Sacramento VA Medical Center
	Tran	Michelle T.	B.S.	Sacramento VA Medical Center
	Barrett	Thomas W.	M.D., M.C.R.	Portland VA Health Care System
	Johnston	Tera	B.S.	Portland VA Health Care System
	Huggins	John T.	M.D.	Charleston VA Medical Center

	Beiko	Tatsiana Y.	M.D.	Charleston VA Medical Center
	Hughes	Heather Y.	M.D.	Charleston VA Medical Center
	McManigle	William C.	M.D.	Charleston VA Medical Center
	Tanner	Nichole T.	M.D.	Charleston VA Medical Center
	Washburn	Ronald G.	M.D.	Charleston VA Medical Center
	Ardelt	Magdalena	A.L.M.	Charleston VA Medical Center
	Tuohy	Patricia A.	B.S.	Charleston VA Medical Center
	Mixson	Jennifer L.	RPh.	Charleston VA Medical Center
	Hinton	Charles G.	PharmD.	Charleston VA Medical Center
	Thornley	Nicola	M.P.H.	Charleston VA Medical Center
	Allen	Heather	R.N.	Charleston VA Medical Center
	Elam	Shannon	R.N.	Charleston VA Medical Center
	Boatman	Barry	R.N., O.C.N.	Charleston VA Medical Center
	Baber	Brittany J.		Charleston VA Medical Center
	Ryant	Rudell	M.B.A	Charleston VA Medical Center
	Roller	Brentin	D.O.	Southern Arizona VA Health Care System
	Nguyen	Chinh	M.D.	Southern Arizona VA Health Care System
	Mikail	Amani Morgan	M.S.C.R.M.	Southern Arizona VA Health Care System
	Hansen	Marivic	Research R.N.	Southern Arizona VA Health Care System
	Lichtenberger	Paola	M.D.	Miami Bruce Carter VA Health CareSystem
	Baracco	Gio	M.D.	Miami Bruce Carter VA Health CareSystem
	Ramos	Carol	M.D.	Miami Bruce Carter VA Health CareSystem
	Bjork	Lauren	PharmD.	Miami Bruce Carter VA Health CareSystem
	Sueiro	Melyssa	M.Sc.	Miami Bruce Carter VA Health CareSystem
	Tien	Phyllis	M.D.	San Francisco VA Health Care System
	Freasier	Heather	M.Sc., R.D.	San Francisco VA Health Care System
	Buck	Theresa	M.D.	Bay Pines VA Healthcare System
	Nekach	Hafida	M.D.	Bay Pines VA Healthcare System
	Holodniy	Mark	M.D., F.A.C.P., F.I.D.S.A.	Veterans Affairs Palo Alto Health Care System
	Chary	Aarthi	M.D.	Veterans Affairs Palo Alto Health Care System
	Lu	Kan	PharmD.	Veterans Affairs Palo Alto Health Care System
	Peters	Theresa	R.N., M.S., C.C.R.C.	Veterans Affairs Palo Alto Health Care System
	Lopez	Jessica	C.C.R.C.	Veterans Affairs Palo Alto Health Care System
	Tan	Susanna Yu	M.D.	VA Long Beach Healthcare System
	Lee	Robert H.	M.D.	VA Long Beach Healthcare System
	Asghar	Aliya	M.P.H	VA Long Beach Healthcare System
	Isip	Tasadduq Karim Karyn	B.A.	VA Long Beach Healthcare System
	Le	Katherine	PharmD.	VA Long Beach Healthcare System
	Nguyen	Thao	PharmD.	VA Long Beach Healthcare System
	Wong	Shinn	PharmD.	VA Long Beach Healthcare System
**CPH ICC**	Raben	Dorthe	M.Sc.	INSIGHT Copenhagen ICC, CHIP (Centre of Excellence for Health, Immunity and Infections), Rigshospitalet, University of Copenhagen, Copenhagen, Denmark
	Murray	Daniel D.	Ph.D.	INSIGHT Copenhagen ICC, CHIP (Centre of Excellence for Health, Immunity and Infections), Rigshospitalet, University of Copenhagen, Copenhagen, Denmark
	Jensen	Tomas O.	M.D.	INSIGHT Copenhagen ICC, CHIP (Centre of Excellence for Health, Immunity and Infections), Rigshospitalet, University of Copenhagen, Copenhagen, Denmark

Peters	Lars	M.D., Ph.D., D.M.Sc.	INSIGHT Copenhagen ICC, CHIP (Centre of Excellence for Health, Immunity and Infections), Rigshospitalet, University of Copenhagen, Copenhagen, Denmark
Aagaard	Bitten	B.Sc.N.	INSIGHT Copenhagen ICC, CHIP (Centre of Excellence for Health, Immunity and Infections), Rigshospitalet, University of Copenhagen, Copenhagen, Denmark
Nielsen	Charlotte B.		INSIGHT Copenhagen ICC, CHIP (Centre of Excellence for Health, Immunity and Infections), Rigshospitalet, University of Copenhagen, Copenhagen, Denmark
Krapp	Katharina	Ph.D.	INSIGHT Copenhagen ICC, CHIP (Centre of Excellence for Health, Immunity and Infections), Rigshospitalet, University of Copenhagen, Copenhagen, Denmark
Nykjær	Bente Rosdahl		INSIGHT Copenhagen ICC, CHIP (Centre of Excellence for Health, Immunity and Infections), Rigshospitalet, University of Copenhagen, Copenhagen, Denmark
Olsson	Christina		INSIGHT Copenhagen ICC, CHIP (Centre of Excellence for Health, Immunity and Infections), Rigshospitalet, University of Copenhagen, Copenhagen, Denmark
Kanne	Katja Lisa	M.Sc., B.Sc.N.	INSIGHT Copenhagen ICC, CHIP (Centre of Excellence for Health, Immunity and Infections), Rigshospitalet, University of Copenhagen, Copenhagen, Denmark
Grevsen	Anne Louise	M.Sc., Dent.	INSIGHT Copenhagen ICC, CHIP (Centre of Excellence for Health, Immunity and Infections), Rigshospitalet, University of Copenhagen, Copenhagen, Denmark
Joensen	Zillah Maria	B.Sc.N.	INSIGHT Copenhagen ICC, CHIP (Centre of Excellence for Health, Immunity and Infections), Rigshospitalet, University of Copenhagen, Copenhagen, Denmark
Bruun	Tina	B.Sc.N.	INSIGHT Copenhagen ICC, CHIP (Centre of Excellence for Health, Immunity and Infections), Rigshospitalet, University of Copenhagen, Copenhagen, Denmark
Bojesen	Ane		INSIGHT Copenhagen ICC, CHIP (Centre of Excellence for Health, Immunity and Infections), Rigshospitalet, University of Copenhagen, Copenhagen, Denmark
Woldbye	Frederik		INSIGHT Copenhagen ICC, CHIP (Centre of Excellence for Health, Immunity and Infections), Rigshospitalet, University of Copenhagen, Copenhagen, Denmark
Normand,	Nick E.	B.Sc.	INSIGHT Copenhagen ICC, CHIP (Centre of Excellence for Health, Immunity and Infections), Rigshospitalet, University of Copenhagen, Copenhagen, Denmark
Esman	Frederik V.L.	B.Sc.	INSIGHT Copenhagen ICC, CHIP (Centre of Excellence for Health, Immunity and Infections), Rigshospitalet, University of Copenhagen, Copenhagen, Denmark
Benfield	Thomas	M.D, D.M.Sc.	Denmark Copenhagen University Hospital - Amager and Hvidovre, Center of Research & Disruption of Infectious Diseases, Department of Infectious Diseases
Clausen	Clara Lundetoft	M.D.	Denmark Copenhagen University Hospital - Amager and Hvidovre, Center of Research & Disruption of Infectious Diseases, Department of Infectious Diseases
Hovmand	Nichlas	M.D.	Denmark Copenhagen University Hospital - Amager and Hvidovre, Center of Research & Disruption of Infectious Diseases, Department of Infectious Diseases
Israelsen	Simone Bastrup	M.D.	Denmark Copenhagen University Hospital - Amager and Hvidovre, Center of Research & Disruption of Infectious Diseases, Department of Infectious Diseases
Iversen	Katrine	M.D.	Denmark Copenhagen University Hospital - Amager and Hvidovre, Center of Research & Disruption of Infectious Diseases, Department of Infectious Diseases
Leding	Caecilie	M.D.	Denmark Copenhagen University Hospital - Amager and Hvidovre, Center of Research & Disruption of Infectious Diseases, Department of Infectious Diseases

Pedersen	Karen Brorup	M.D.	Denmark Copenhagen University Hospital - Amager and Hvidovre, Center of Research & Disruption of Infectious Diseases, Department of Infectious Diseases
Thorlacius-Ussing	Louise	M.D.	Denmark Copenhagen University Hospital - Amager and Hvidovre, Center of Research & Disruption of Infectious Diseases, Department of Infectious Diseases
Tinggaard	Michaela	M.D.	Denmark Copenhagen University Hospital - Amager and Hvidovre, Center of Research & Disruption of Infectious Diseases, Department of Infectious Diseases
Tingsgard	Sandra	M.D.	Denmark Copenhagen University Hospital - Amager and Hvidovre, Center of Research & Disruption of Infectious Diseases, Department of Infectious Diseases
Krohn-Dehli	Louise	R.N.	Denmark Copenhagen University Hospital - Amager and Hvidovre, Center of Research & Disruption of Infectious Diseases, Department of Infectious Diseases
Pedersen	Dorthe	R.N.	Denmark Copenhagen University Hospital - Amager and Hvidovre, Center of Research & Disruption of Infectious Diseases, Department of Infectious Diseases
Villadsen	Signe	R.N.	Denmark Copenhagen University Hospital - Amager and Hvidovre, Center of Research & Disruption of Infectious Diseases, Department of Infectious Diseases
Jensen	Jens-Ulrik Staehr	M.D., Ph.D.	Herlev-Gentofte Hospital, Respiratory Medicine Section, Department of Internal Medicine
Overgaard	Rikke	R.N.	Herlev-Gentofte Hospital, Respiratory Medicine Section, Department of Internal Medicine
Rastoder	Ema	M.D.	Herlev-Gentofte Hospital, Respiratory Medicine Section, Department of Internal Medicine
Heerfordt	Christian	M.D.	Herlev-Gentofte Hospital, Respiratory Medicine Section, Department of Internal Medicine
Hedsund	Caroline	M.D	Herlev-Gentofte Hospital, Respiratory Medicine Section, Department of Internal Medicine
Ronn	Christian Phillip	M.D.	Herlev-Gentofte Hospital, Respiratory Medicine Section, Department of Internal Medicine
Kamstrup	Peter Thobias	M.D.	Herlev-Gentofte Hospital, Respiratory Medicine Section, Department of Internal Medicine
Hogsberg	Dorthe Sandbaek	R.N.	Herlev-Gentofte Hospital, Respiratory Medicine Section, Department of Internal Medicine
Bergsoe	Christina	B.Sc.	Herlev-Gentofte Hospital, Respiratory Medicine Section, Department of Internal Medicine
Søborg	Christian	M.D., Ph.D.	Herlev-Gentofte Hospital, Respiratory Medicine Section, Department of Internal Medicine
Hissabu	Nuria M.S.	B.Sc.	Herlev-Gentofte Hospital, Respiratory Medicine Section, Department of Internal Medicine
Arp	Bodil C.	B.Sc.	Herlev-Gentofte Hospital, Respiratory Medicine Section, Department of Internal Medicine
Ostergaard	Lars	M.D., Ph.D., D.M.Sc.	Aarhus Universitetshospital, Skejby
Staerke	Nina Breinholt	M.D.	Aarhus Universitetshospital, Skejby
Yehdego	Yordanos	R.N.	Aarhus Universitetshospital, Skejby
Sondergaard	Ane	R.N.	Aarhus Universitetshospital, Skejby
Johansen	Isik S.	M.D., D.M.Sc.	Odense University Hospital, Department of Infectious Diseases
Arnholdt Pedersen	Andreas	M.D.	Odense University Hospital, Department of Infectious Diseases
Knudtzen	Fredrikke C.	M.D.	Odense University Hospital, Department of Infectious Diseases
Larsen	Lykke	M.D.	Odense University Hospital, Department of Infectious Diseases
Hertz	Mathias A.	M.D.	Odense University Hospital, Department of Infectious Diseases

Fabricius	Thilde	M.D.	Odense University Hospital, Department of Infectious Diseases
Holden	Inge K.	M.D., Ph.D.	Odense University Hospital, Department of Infectious Diseases
Lindvig	Susan O.	M.Sc.	Odense University Hospital, Department of Infectious Diseases
Helleberg	Marie	M.D., Ph.D., D.M.Sc.	Dept. of Infectious Diseases, Rigshospitalet, Copenhagen University Hospital
Gerstoft	Jan	M.D., D.M.Sc.	Dept. of Infectious Diseases, Rigshospitalet, Copenhagen University Hospital
Kirk	Ole	M.D., D.M.Sc.	Dept. of Infectious Diseases, Rigshospitalet, Copenhagen University Hospital
Bruun	Tina	R.N., M.Sc., PH.	Dept. of Infectious Diseases, Rigshospitalet, Copenhagen University Hospital
Jensen	Tomas Ostergaard	M.D.	North Zealand University Hospital, Department of Pulmonary and Infectious Diseases
Madsen	Birgitte Lindegaard	M.D.	North Zealand University Hospital, Department of Pulmonary and Infectious Diseases
Pedersen	Thomas Ingemann	M.D.	North Zealand University Hospital, Department of Pulmonary and Infectious Diseases
Harboe	Zitta Barrella	M.D.	North Zealand University Hospital, Department of Pulmonary and Infectious Diseases
Roge	Birgit Thorup	M.D., Ph.D.	Kolding Hospital, Department of Medicine
Hansen	Thomas Michael	M.D.	Kolding Hospital, Department of Medicine
Glesner	Matilde Kanstrup	M.D.	Kolding Hospital, Department of Medicine
Lofberg	Sandra Valborg	M.D.	Kolding Hospital, Department of Medicine
Nielsen	Ariella Denize	M.D.	Kolding Hospital, Department of Medicine
Leicht von Huth	Sebastian	M.D., Ph.D.	Kolding Hospital, Department of Medicine
Nielsen	Henrik	M.D., D.M.Sci.	Aalborg University Hospital, Department of Infectious Diseases
Thisted	Rikke Krog	R.N.	Aalborg University Hospital, Department of Infectious Diseases
Petersen	Kristine Toft	R.N.	Aalborg University Hospital, Department of Infectious Diseases
Juhl	Maria Ruwald	R.N	Aalborg University Hospital, Department of Infectious Diseases
Podlekareva	Daria	M.D., Ph.D.	Department of Respiratory Medicine, Bispebjerg Hospital, Copenhagen, Denmark
Johnsen	Stine	M.D., Ph.D.	Department of Respiratory Medicine, Bispebjerg Hospital, Copenhagen, Denmark
Andreassen	Helle Frost	M.D., Ph.D.	Department of Respiratory Medicine, Bispebjerg Hospital, Copenhagen, Denmark
Pedersen	Lars	M.D., Ph.D.	Department of Respiratory Medicine, Bispebjerg Hospital, Copenhagen, Denmark
Lindnér	Cecilia Ebba Clara Ellinor	M.D.	Department of Respiratory Medicine, Bispebjerg Hospital, Copenhagen, Denmark
Wiese	Lothar	M.D., Ph.D.	Department of Infectious Diseases, Zealand University Hospital Roskilde and Department of Internal Medicine, Zealand University Hospital Koge
Knudsen	Lene Surland	M.D., Ph.D.	Department of Infectious Diseases, Zealand University Hospital Roskilde and Department of Internal Medicine, Zealand University Hospital Koge
Nytofte	Nikolaj Julian Skrøder	M.D., Ph.D.	Department of Infectious Diseases, Zealand University Hospital Roskilde and Department of Internal Medicine, Zealand University Hospital Koge

Havmøller	Signe Ravn	M.D.	Department of Infectious Diseases, Zealand University Hospital Roskilde and Department of Internal Medicine, Zealand University Hospital Koge
Expósito	Maria	B.Sc.	Spain INSIGHT SCC Spain, Hospital Universitari Germans Trias i Pujol, Badalona
Badillo	José	B.Sc.	Spain INSIGHT SCC Spain, Hospital Universitari Germans Trias i Pujol, Badalona
Martínez	Ana	B.Sc.	Spain INSIGHT SCC Spain, Hospital Universitari Germans Trias i Pujol, Badalona
Abad	Elena	B.Sc.	Spain INSIGHT SCC Spain, Hospital Universitari Germans Trias i Pujol, Badalona
Chamorro	Ana	B.Sc.	Spain INSIGHT SCC Spain, Hospital Universitari Germans Trias i Pujol, Badalona
Figuerola	Ariadna	B.Sc.	Spain INSIGHT SCC Spain, Hospital Universitari Germans Trias i Pujol, Badalona
Mateu	Lourdes	M.D., Ph.D.	Hospital Universitari Germans Trias i Pujol, Badalona
España	Sergio	M.D.	Hospital Universitari Germans Trias i Pujol, Badalona
Lucero	Maria Constanza	MD., Ph.D.	Hospital Universitari Germans Trias i Pujol, Badalona
Santos	José Ramón	M.D., Ph.D.	Hospital Universitari Germans Trias i Pujol, Badalona
Lladós	Gemma	M.D.	Hospital Universitari Germans Trias i Pujol, Badalona
Lopez	Cristina	M.D., Ph.D.	Hospital Universitari Germans Trias i Pujol, Badalona
Carabias	Lydia	M.D.	Hospital Universitari Germans Trias i Pujol, Badalona
Molina-Morant	Daniel	M.D., Ph.D.	Hospital Universitari Germans Trias i Pujol, Badalona
Loste	Cora	M.D., Ph.D.	Hospital Universitari Germans Trias i Pujol, Badalona
Bracke	Carmen	M.D.	Hospital Universitari Germans Trias i Pujol, Badalona
Siles	Adrian	B.Sc.	Hospital Universitari Germans Trias i Pujol, Badalona
Fernández-Cruz	Eduardo	M.D., Ph.D.	Hospital General Universitario Gregorio Marañón, Madrid
Natale	Marisa Di	M.D.	Hospital General Universitario Gregorio Marañón, Madrid
Padure	Sergiu		Hospital General Universitario Gregorio Marañón, Madrid
Gomez	Jimena	M.D.	Hospital General Universitario Gregorio Marañón, Madrid
Ausin	Cristina	M.D.	Hospital General Universitario Gregorio Marañón, Madrid
Cervilla	Eva	M.D.	Hospital General Universitario Gregorio Marañón, Madrid
Balastegui	Héctor	M.D.	Hospital General Universitario Gregorio Marañón, Madrid
Sainz	Carmen Rodríguez	Ph.D.	Hospital General Universitario Gregorio Marañón, Madrid
Lopez	Paco	M.D.	Hospital General Universitario Gregorio Marañón, Madrid
Carbone	Javier	M.D., Ph.D.	Hospital General Universitario Gregorio Marañón, Madrid
Escobar	Mariam	R.N.	Hospital General Universitario Gregorio Marañón, Madrid
Balerdi	Leire	M.D.	Barcelona Institute for Global Health (ISGlobal), Hospital Clínic - Universitat de Barcelona, Barcelona
Legarda	Almudena		Barcelona Institute for Global Health (ISGlobal), Hospital Clínic - Universitat de Barcelona, Barcelona
Roldan	Montserrat		Barcelona Institute for Global Health (ISGlobal), Hospital Clínic - Universitat de Barcelona, Barcelona
Letona	Laura	M.D.	Barcelona Institute for Global Health (ISGlobal), Hospital Clínic - Universitat de Barcelona, Barcelona
Muñoz	José	M.D., Ph.D.	Barcelona Institute for Global Health (ISGlobal), Hospital Clínic - Universitat de Barcelona, Barcelona
Camprubí	Daniel	M.D.	Barcelona Institute for Global Health (ISGlobal), Hospital Clínic - Universitat de Barcelona, Barcelona
Arribas	Jose R.	M.D.	Hospital Universitario La Paz, IdiPAZ, Madrid
Sánchez	Rocio Montejano	M.D., Ph.D.	Hospital Universitario La Paz, IdiPAZ, Madrid
Díaz-Pollán	Beatriz	M.D., Ph.D.	Hospital Universitario La Paz, IdiPAZ, Madrid
Stewart	Stefan Mark	M.D.	Hospital Universitario La Paz, IdiPAZ, Madrid

Garcia	Irene	M.D.	Hospital Universitario La Paz, IdiPAZ, Madrid
Borobia	Alberto	M.D., Ph.D.	Hospital Universitario La Paz, IdiPAZ, Madrid
Mora-Rillo	Marta	M.D., Ph.D.	Hospital Universitario La Paz, IdiPAZ, Madrid
Estrada	Vicente	M.D., Ph.D.	Hospital Clínico San Carlos, Madrid
Cabello	Noemi	M.D.	Hospital Clínico San Carlos, Madrid
Nuñez-Orantos	M.J.	M.D.	Hospital Clínico San Carlos, Madrid
Sagastagoitia	I.	M.D.	Hospital Clínico San Carlos, Madrid
Homen	J.R.	MD	Hospital Clínico San Carlos, Madrid
Orviz	E.	MD.	Hospital Clínico San Carlos, Madrid
Montalvá	Adrián Sánchez	M.D., P.hD.	Hospital Universitary Vall d'Hebron, Barcelona
Espinosa-Pereiro	Juan	M.D.	Hospital Universitary Vall d'Hebron, Barcelona
Bosch-Nicolau	Pau	M.D.	Hospital Universitary Vall d'Hebron, Barcelona
Salvador	Fernando	M.D., P.hD.	Hospital Universitary Vall d'Hebron, Barcelona
Burgos	Joaquin	M.D., Ph.D.	Hospital Universitary Vall d'Hebron, Barcelona
Morales-Rull	Jose Luis	M.D., Ph.D.	Internal Medicine Department. University Hospital Arnau de Vilanova, Lleida
Pena	Anna Maria Moreno	M.D.	Internal Medicine Department. University Hospital Arnau de Vilanova, Lleida
Acosta	Cristina	M.D.	Internal Medicine Department. University Hospital Arnau de Vilanova, Lleida
Solé-Felip	Cristina	M.D.	Internal Medicine Department. University Hospital Arnau de Vilanova, Lleida
Horcajada	Juan P.	M.D., Ph.D.	Hospital del Mar, Barcelona
Sendra	Elena	M.D.	Hospital del Mar, Barcelona
Castañeda	Silvia	M.D.	Hospital del Mar, Barcelona
López-Montesinos	Inmaculada	M.D.	Hospital del Mar, Barcelona
Gómez-Junyent	Joan	M.D.	Hospital del Mar, Barcelona
Gonzáles	Carlota Gudiol	M.D.	Infectious Diseases Department, Bellvitge University Hospital, IDIBELL, University of Barcelona, CIBERINFEC
Cuervo	Guilermo	M.D., Ph.D.	Infectious Diseases Department, Bellvitge University Hospital, IDIBELL, University of Barcelona, CIBERINFEC
Pujol	Miquel	M.D., Ph.D.	Infectious Diseases Department, Bellvitge University Hospital, IDIBELL, University of Barcelona, CIBERINFEC
Carratalà	Jordi	M.D., Ph.D.	Infectious Diseases Department, Bellvitge University Hospital, IDIBELL, University of Barcelona, CIBERINFEC
Videla	Sebastià	M.D., Ph.D.	Infectious Diseases Department, Bellvitge University Hospital, IDIBELL, University of Barcelona, CIBERINFEC
Günthard	Huldrych	M.D.	Switzerland Department of Infectious Diseases and Hospital Epidemiology, University Hospital Zurich and Institute of Medical Virology, University of Zurich, Zurich Switzerland
Braun	Dominique L.	M.D.	Switzerland Department of Infectious Diseases and Hospital Epidemiology, University Hospital Zurich and Institute of Medical Virology, University of Zurich, Zurich Switzerland
West	Emily	M.D.	Switzerland Department of Infectious Diseases and Hospital Epidemiology, University Hospital Zurich and Institute of Medical Virology, University of Zurich, Zurich Switzerland
M’Rabeth-Bensalah	Khadija	M.D.	Switzerland Department of Infectious Diseases and Hospital Epidemiology, University Hospital Zurich and Institute of Medical Virology, University of Zurich, Zurich Switzerland
Eichinger	Mareile L.	M.D.	Switzerland Department of Infectious Diseases and Hospital Epidemiology, University Hospital Zurich and Institute of Medical Virology, University of Zurich, Zurich Switzerland

Grüttner-Durmaz	Manuela	R.N.	Switzerland Department of Infectious Diseases and Hospital Epidemiology, University Hospital Zurich and Institute of Medical Virology, University of Zurich, Zurich Switzerland
Grube	Christina	R.N.	Switzerland Department of Infectious Diseases and Hospital Epidemiology, University Hospital Zurich and Institute of Medical Virology, University of Zurich, Zurich Switzerland
Zink	Veronika	M.Sc., pharmacist	Switzerland Department of Infectious Diseases and Hospital Epidemiology, University Hospital Zurich and Institute of Medical Virology, University of Zurich, Zurich Switzerland
Goes	Josefine	pharmacist	Switzerland Department of Infectious Diseases and Hospital Epidemiology, University Hospital Zurich and Institute of Medical Virology, University of Zurich, Zurich Switzerland
Fätkenheuer	Gerd	M.D.	Department I of Internal Medicine, Division of Infectious Diseases, University of Cologne, Germany
Malin	Jakob J.	M.D.	Department I of Internal Medicine, Division of Infectious Diseases, University of Cologne, Germany
Tsertsvadze	Tengiz	M.D., Ph.D.	Georgia SCC, Infectious Diseases, AIDS and Clinical Immunology Research Center, Tbilisi, Georgia
Abutidze	Akaki	M.D., M.P.H., Ph.D.	Georgia SCC, Infectious Diseases, AIDS and Clinical Immunology Research Center, Tbilisi, Georgia
Chkhartishvili	Nikoloz	M.D., M.S., Ph.D.	Georgia SCC, Infectious Diseases, AIDS and Clinical Immunology Research Center, Tbilisi, Georgia
Metchurtchlishvili	Revaz	M.D.	Georgia SCC, Infectious Diseases, AIDS and Clinical Immunology Research Center, Tbilisi, Georgia
Endeladze	Marina	M.D.	Georgia SCC, Infectious Diseases, AIDS and Clinical Immunology Research Center, Tbilisi, Georgia
Paciorek	Marcin	M.D., Ph.D.	Poland SCC, Wojewodzki Szpital Zakazny Warsaw
Bursa	Dominik	M.D., Ph.D.	Poland SCC, Wojewodzki Szpital Zakazny Warsaw
Krogulec	Dominika	M.D.	Poland SCC, Wojewodzki Szpital Zakazny Warsaw
Pulik	Piotr	M.D.	Poland SCC, Wojewodzki Szpital Zakazny Warsaw
Ignatowska	Anna	M.D.	Poland SCC, Wojewodzki Szpital Zakazny Warsaw
Horban	Andrzej	M.D., Ph.D.	Poland SCC, Wojewodzki Szpital Zakazny Warsaw
Bakowska	Elzbieta	M.D.	Poland SCC, Wojewodzki Szpital Zakazny Warsaw
Kowaska	Justyna	M.D., Ph.D.	Poland SCC, Wojewodzki Szpital Zakazny Warsaw
Bednarska	Agnieszka	M.D., Ph.D.	Poland SCC, Wojewodzki Szpital Zakazny Warsaw
Jurek	Natalia	M.D.	Poland SCC, Wojewodzki Szpital Zakazny Warsaw
Skrzat-Klapaczynska	Agata	M.D., Ph.D.	Poland SCC, Wojewodzki Szpital Zakazny Warsaw
Bienkowski	Carlo	M.D.	Poland SCC, Wojewodzki Szpital Zakazny Warsaw
Hackiewicz	Malgorzata	M.D.	Poland SCC, Wojewodzki Szpital Zakazny Warsaw
Makowiecki	Michal	M.D.	Poland SCC, Wojewodzki Szpital Zakazny Warsaw
Platowski	Antoni	M.D.	Poland SCC, Wojewodzki Szpital Zakazny Warsaw
Fishchuk	Roman	M.D	Ukraine Central City Clinical Hospital of Ivano-Frankivsk City, Ukraine
Kobrynska	Olena	M.D	Ukraine Central City Clinical Hospital of Ivano-Frankivsk City, Ukraine
Levandovska	Khrystyna	M.D	Ukraine Central City Clinical Hospital of Ivano-Frankivsk City, Ukraine
Kirieieva	Ivanna		Ukraine Central City Clinical Hospital of Ivano-Frankivsk City, Ukraine
Kuziuk	Mykhailo		Ukraine Central City Clinical Hospital of Ivano-Frankivsk City, Ukraine
Naucler	Pontus	M.D., Ph.D.	Sweden, Dept. Of Infectious Diseases, Karolinska University Hospital and Division of Infectious Diseases, Dept. Of Medicine, Solna, Karolinska Instituttet

	Perlhamre	Emma	M.Sc.	Sweden, Dept. Of Infectious Diseases, Karolinska University Hospital and Division of Infectious Diseases, Dept. Of Medicine, Solna, Karolinska Instituttet
	Mazouch	Lotta	M.Sc.	Sweden, Dept. Of Infectious Diseases, Karolinska University Hospital and Division of Infectious Diseases, Dept. Of Medicine, Solna, Karolinska Instituttet
**SYD ICC**	Kelleher	Anthony	M.B.B.S., Ph.D.	INSIGHT Sydney ICC, The Kirby Institute, University of New South Wales, Sydney, Australia
	Polizzotto	Mark	M.D., Ph.D.	INSIGHT Sydney ICC, The Kirby Institute, University of New South Wales, Sydney, Australia
	Carey	Catherine	B.A., M.Sc.	INSIGHT Sydney ICC, The Kirby Institute, University of New South Wales, Sydney, Australia
	Chang	Christina C.	M.D., Ph.D.	INSIGHT Sydney ICC, The Kirby Institute, University of New South Wales, Sydney, Australia
	Hough	Sally	B.Sc.	INSIGHT Sydney ICC, The Kirby Institute, University of New South Wales, Sydney, Australia
	Virachit	Sophie	B.Sc., Ph.D.	INSIGHT Sydney ICC, The Kirby Institute, University of New South Wales, Sydney, Australia
	Davidson	Sarah	B.N.	INSIGHT Sydney ICC, The Kirby Institute, University of New South Wales, Sydney, Australia
	Bice	Daniel J.	B.MSc.	INSIGHT Sydney ICC, The Kirby Institute, University of New South Wales, Sydney, Australia
	Ognenovska	Katherine	B.Sc., Ph.D.	INSIGHT Sydney ICC, The Kirby Institute, University of New South Wales, Sydney, Australia
	Cabrera	Gesalit	B.MSc., M.I.P.H.	INSIGHT Sydney ICC, The Kirby Institute, University of New South Wales, Sydney, Australia
	Flynn	Ruth	B.App.Sc., M.App.Sc.	INSIGHT Sydney ICC, The Kirby Institute, University of New South Wales, Sydney, Australia
	Young	Barnaby E.	M.B.B.S., Ph.D.	Tan Tock Seng Hospital, NCID, Singapore (21 participants enrolled)
	Chia	Po Ying	M.B.B.S.	Tan Tock Seng Hospital, NCID, Singapore (21 participants enrolled)
	Lee	Tau Hong	M.B.B.S.	Tan Tock Seng Hospital, NCID, Singapore (21 participants enrolled)
	Lin	Ray J.	M.B.B.S.	Tan Tock Seng Hospital, NCID, Singapore (21 participants enrolled)
	Lye	David C.	M.B.B.S.	Tan Tock Seng Hospital, NCID, Singapore (21 participants enrolled)
	Ong	Sean W.X.	M.B.B.S.	Tan Tock Seng Hospital, NCID, Singapore (21 participants enrolled)
	Puah	Ser Hon	M.B.B.S.	Tan Tock Seng Hospital, NCID, Singapore (21 participants enrolled)
	Yeo	Tsin Wen	M.B.B.S., Ph.D.	Tan Tock Seng Hospital, NCID, Singapore (21 participants enrolled)
	Diong	Shiau Hui	B.Bio., M.Sc.	Tan Tock Seng Hospital, NCID, Singapore (21 participants enrolled)
	Ongko	Juwinda	B.Sc.	Tan Tock Seng Hospital, NCID, Singapore (21 participants enrolled)
	Yeo	He Ping	B.Sc.	Tan Tock Seng Hospital, NCID, Singapore (21 participants enrolled)
	Eriobu	Nnakelu	M.D., M.P.H.	Institute of Human Virology-Nigeria (IHVN) (9 participants enrolled)
	Kwaghe	Vivian	M.D.	Institute of Human Virology-Nigeria (IHVN) (9 participants enrolled)
	Zaiyad	Habib	M.D.	Institute of Human Virology-Nigeria (IHVN) (9 participants enrolled)
	Idoko	Godwin	M.D.	Institute of Human Virology-Nigeria (IHVN) (9 participants enrolled)
	Uche	Blessing	R.M., R.N.	Institute of Human Virology-Nigeria (IHVN) (9 participants enrolled)

	Selvamuthu	Poongulali	M.B.B.S., M.Sc., Ph.D.	Chennai Antiviral Research and Treatment Clinical Research Site, India
	Kumarasamy	Nagalingeswaran	M.B.B.S., Ph.D.	Chennai Antiviral Research and Treatment Clinical Research Site, India
	Beulah	Faith Ester	B.Sc., M.A., M.Sc.	Chennai Antiviral Research and Treatment Clinical Research Site, India
	Govindarajan	Narayan	B.Pharm.	Chennai Antiviral Research and Treatment Clinical Research Site, India
	Mariyappan	Kowsalya	B.Pharm.	Chennai Antiviral Research and Treatment Clinical Research Site, India
	Losso	Marcelo H.	M.D., M.S.	INSIGHT SCC Argentina, Coordinación en Investigación Clínica Académica en Latinoamérica
	Abela	Cecilia	R.N., B.S.N.	INSIGHT SCC Argentina, Coordinación en Investigación Clínica Académica en Latinoamérica
	Moretto	Renzo	M.D.	INSIGHT SCC Argentina, Coordinación en Investigación Clínica Académica en Latinoamérica
	Belloc	Carlos G.	B.Sc., Ph.D.	INSIGHT SCC Argentina, Coordinación en Investigación Clínica Académica en Latinoamérica
	Ludueña	Jael	T.B.A.	INSIGHT SCC Argentina, Coordinación en Investigación Clínica Académica en Latinoamérica
	Amar	Josefina		INSIGHT SCC Argentina, Coordinación en Investigación Clínica Académica en Latinoamérica
	Losso	Marcelo H.	M.D., M.S.	Hospital General de Agudos JM Ramos Mejia, Buenos Aires
	Toibaro	Javier	M.D., B.C.	Hospital General de Agudos JM Ramos Mejia, Buenos Aires
	Macias	Laura Moreno	M.D.	Hospital General de Agudos JM Ramos Mejia, Buenos Aires
	Fernandez	Lucia	M.D.	Hospital General de Agudos JM Ramos Mejia, Buenos Aires
	Frare	Pablo S.	M.D.	Hospital General de Agudos JM Ramos Mejia, Buenos Aires
	Chaio	Sebastian R.	M.D.	Hospital General de Agudos JM Ramos Mejia, Buenos Aires
	Pachioli	Valeria	M.D.	Hospital General de Agudos JM Ramos Mejia, Buenos Aires
	Timpano	Stella M.	B.Pharm.	Hospital General de Agudos JM Ramos Mejia, Buenos Aires
	Sanchez	Marisa del Lujan	M.D.	Hospital Italiano de Buenos Aires, Buenos Aires
	Sierra	Mariana de Paz	M.D.	Hospital Italiano de Buenos Aires, Buenos Aires
	Stanek	Vanina	M.D.	Hospital Italiano de Buenos Aires, Buenos Aires
	Belloso	Waldo	M.D.	Hospital Italiano de Buenos Aires, Buenos Aires
	Cilenti	Flavia L.		Hospital Italiano de Buenos Aires, Buenos Aires
	Valentini	Ricardo N.	M.D.	Centro de Educacion Medica e Investigaciones Clinicas, Buenos Aires
	Stryjewski	Martin E.	M.D.	Centro de Educacion Medica e Investigaciones Clinicas, Buenos Aires
	Locatelli	Nicolas	M.D.	Centro de Educacion Medica e Investigaciones Clinicas, Buenos Aires
	Soler Riera	Maria C.	M.D.	Centro de Educacion Medica e Investigaciones Clinicas, Buenos Aires
	Salgado	Clara	M.D.	Centro de Educacion Medica e Investigaciones Clinicas, Buenos Aires
	Baeck	Ines M.	M.D.	Centro de Educacion Medica e Investigaciones Clinicas, Buenos Aires
	Di Castelnuovo	Valentina	M.D.	Centro de Educacion Medica e Investigaciones Clinicas, Buenos Aires
	Zarza	Stella M.	M.D.	Centro de Educacion Medica e Investigaciones Clinicas, Buenos Aires
**LON ICC**	Hudson	Fleur	B.A.	INSIGHT London ICC, MRC Clinical Trials Unit at UC, London, UK
	Parmar	Mahesh K.B.	Ph.D.	INSIGHT London ICC, MRC Clinical Trials Unit at UC, London, UK
	Goodman	Anna L.	F.R.C.P., Dphil	INSIGHT London ICC, MRC Clinical Trials Unit at UC, London, UK

Badrock	Jonathan	B.Sc.	INSIGHT London ICC, MRC Clinical Trials Unit at UC, London, UK
Gregory	Adam	M.A.	INSIGHT London ICC, MRC Clinical Trials Unit at UC, London, UK
Goodall	Katharine	B.A.	INSIGHT London ICC, MRC Clinical Trials Unit at UC, London, UK
Harris	Nicola		INSIGHT London ICC, MRC Clinical Trials Unit at UC, London, UK
Wyncoll	James	B.Sc.	INSIGHT London ICC, MRC Clinical Trials Unit at UC, London, UK
Bhagani	S.	M.D.	United Kingdom SCC: Royal Free Hospital
Rodger	A.	Ph.D.	United Kingdom SCC: Royal Free Hospital
Luntiel	A.	M.D.	United Kingdom SCC: Royal Free Hospital
Patterson	C.	M.D.	United Kingdom SCC: Royal Free Hospital
Morales	J.	B.Sc	United Kingdom SCC: Royal Free Hospital
Witele	E.	B.Sc.	United Kingdom SCC: Royal Free Hospital
Preston	A-M	B.Sc.	United Kingdom SCC: Royal Free Hospital
Nandani	A.	M.Pharm.	United Kingdom SCC: Royal Free Hospital
Price	D.A.	M.D.	Royal Victoria Infirmary
Hanrath	Aiden	M.B.B.S.	Royal Victoria Infirmary
Nell	Jeremy	M.D.	Royal Victoria Infirmary
Patel	Bijal	M.Sc.	Royal Victoria Infirmary
Hays	Carole	A.D.N.S.	Royal Victoria Infirmary
Jones	Geraldine	B.Sc.	Royal Victoria Infirmary
Davidson	Jade	B.T.E.C. Pharm	Royal Victoria Infirmary
Goodman	Anna L.	F.R.C.P., D.Phil.	Guy’s & St. Thomas’ NHS Foundation Trust
Bawa	T.	M.B.B.S.	Guy’s & St. Thomas’ NHS Foundation Trust
Mathews	M.	M.Sc., B.Pharm.	Guy’s & St. Thomas’ NHS Foundation Trust
Mazzella	A.	M.R.C.P., M.Sc.	Guy’s & St. Thomas’ NHS Foundation Trust
Bisnauthsing	K.	B.Sc.	Guy’s & St. Thomas’ NHS Foundation Trust
Aguilar-Jimenez	L.	B.Sc.	Guy’s & St. Thomas’ NHS Foundation Trust
Borchini	F.	B.Sc.	Guy’s & St. Thomas’ NHS Foundation Trust
Hammett	S.	B.Sc.	Guy’s & St. Thomas’ NHS Foundation Trust
Touloumi	Giota	Ph.D.	Greece SCC, National & Kapodistrian University of Athens Medical School
Pantazis	Nikos	Ph.D.	Greece SCC, National & Kapodistrian University of Athens Medical School
Gioukari	Vicky	B.Sc.	Greece SCC, National & Kapodistrian University of Athens Medical School
Souliou	Tania		Greece SCC, National & Kapodistrian University of Athens Medical School
Antoniadou	A.	M.D	Attikon University General Hospital
Protopapas	K.	M.D.	Attikon University General Hospital
Kavatha	D.	M.D.	Attikon University General Hospital
Grigoropoulou	S.	M.D.	Attikon University General Hospital
Tziolos	R-N.	M.D.	Attikon University General Hospital
Oikonomopoulo	C.		Attikon University General Hospital
Moschopoulos	C.	M.D.	Attikon University General Hospital
Koulouris	N.G.	M.D.	1st Respiratory Medicine Department, Athens University Medical School

Tzimopoulos	K.	M.D.	1st Respiratory Medicine Department, Athens University Medical School
Koromilias	A.	M.D.	1st Respiratory Medicine Department, Athens University Medical School
Argyraki	K.	M.D.	1st Respiratory Medicine Department, Athens University Medical School
Lourida	P.	M.D.	1st Respiratory Medicine Department, Athens University Medical School
Bakakos	P.	M.D.	1st Respiratory Medicine Department, Athens University Medical School
Kalomenidis	I.	M.D.	Department of Critical Care and Pulmonary Medicine, Evangelismos General Hospital
Vlachakos	V.	M.D.	Department of Critical Care and Pulmonary Medicine, Evangelismos General Hospital
Barmparessou	Z.	M.D.	Department of Critical Care and Pulmonary Medicine, Evangelismos General Hospital
Balis	E.	M.D., Ph.D.	Department of Critical Care and Pulmonary Medicine, Evangelismos General Hospital
Zakynthinos	S.	M.D.	Department of Critical Care and Pulmonary Medicine, Evangelismos General Hospital
Sigala	I.	M.D.	Department of Critical Care and Pulmonary Medicine, Evangelismos General Hospital
Gianniou	N.	M.D.	Department of Critical Care and Pulmonary Medicine, Evangelismos General Hospital
Dima	E.	M.D.	Department of Critical Care and Pulmonary Medicine, Evangelismos General Hospital
Magkouta	S.	M.D.	Department of Critical Care and Pulmonary Medicine, Evangelismos General Hospital
Synolaki	E.	M.D.	Department of Critical Care and Pulmonary Medicine, Evangelismos General Hospital
Konstanta	S.	M.D.	Department of Critical Care and Pulmonary Medicine, Evangelismos General Hospital
Vlachou	M.	M.Sc., Ph.D.	Department of Critical Care and Pulmonary Medicine, Evangelismos General Hospital
Stathopoulou	P.	M.Sc.	Department of Critical Care and Pulmonary Medicine, Evangelismos General Hospital
Panagopoulos	P.	MD	Democritus University of Thrace
Petrakis	V.	MD	Democritus University of Thrace
Papazoglou	D.	MD	Democritus University of Thrace
Tompaidou	E.	M.Sc.	Democritus University of Thrace
Isaakidou	E.		Democritus University of Thrace
Poulakou	G.	M.D.	3rd Department of Medicine, Medical School, NKUA
Rapti	V.	M.D.	3rd Department of Medicine, Medical School, NKUA
Leontis	K.	M.D.	3rd Department of Medicine, Medical School, NKUA
Nitsotolis	T.	M.D.	3rd Department of Medicine, Medical School, NKUA
Athanasiou	K.	M.D.	3rd Department of Medicine, Medical School, NKUA
Syrigos	K.	M.D.	3rd Department of Medicine, Medical School, NKUA
Argyraki	K.	M.D.	3rd Department of Medicine, Medical School, NKUA
Myrodia	M-D.	M.D.	3rd Department of Medicine, Medical School, NKUA
Kyriakoulis	K.	M.D.	3rd Department of Medicine, Medical School, NKUA
Trontzas	I.	M.D.	3rd Department of Medicine, Medical School, NKUA
Arfara-Melanini	M.	M.D.	3rd Department of Medicine, Medical School, NKUA
Kolonis	V.	M.D.	3rd Department of Medicine, Medical School, NKUA
Kityo	Cissy	M.D.	Uganda SCC, JCRC/MRC/UVRI Uganda Research Unit
Mugerwa	Henry	M.D.	Uganda SCC, JCRC/MRC/UVRI Uganda Research Unit
Kiweewa	Francis	M.D., M.P.H.	Uganda SCC, JCRC/MRC/UVRI Uganda Research Unit

Kimuli	Ivan	M.D.	Uganda SCC, JCRC/MRC/UVRI Uganda Research Unit
Lukaakome	Joseph	M.D.	MRC/UVRI & LSHTM Uganda Research Unit
Nsereko	Christoher	M.D.	MRC/UVRI & LSHTM Uganda Research Unit
Lubega	Gloria	M.D.	MRC/UVRI & LSHTM Uganda Research Unit
Kibirige	Moses	M.D.	MRC/UVRI & LSHTM Uganda Research Unit
Nakahima	William		MRC/UVRI & LSHTM Uganda Research Unit
Wangi	Deus		MRC/UVRI & LSHTM Uganda Research Unit
Aguti	Evelyne	M.D.	MRC/UVRI & LSHTM Uganda Research Unit
Generous	Lilian		MRC/UVRI & LSHTM Uganda Research Unit
Massa	Rosemary		MRC/UVRI & LSHTM Uganda Research Unit
Nalaki	Margaret		MRC/UVRI & LSHTM Uganda Research Unit
Magala	Felix	M.D.	MRC/UVRI & LSHTM Uganda Research Unit
Nabaggala	Phiona Kaweesi		MRC/UVRI & LSHTM Uganda Research Unit
Kidega	Robert	M.D.	Gulu Regional Referral Hospital
Kityo	Cissy	M.D., Ph.D.	Gulu Regional Referral Hospital
Mugerwa	Henry	M.D.	Gulu Regional Referral Hospital
Faith	Oryem Daizy	R.N.	Gulu Regional Referral Hospital
Florence	Apio	R.N.	Gulu Regional Referral Hospital
Emmanuel	Ocung	B.B.L.T.	Gulu Regional Referral Hospital
Beacham	Mugoonyi Paul	M.D.	Gulu Regional Referral Hospital
Geoffrey	Amone	B.Sc.	Gulu Regional Referral Hospital
Nakiboneka	Dridah	B.Stat.	Gulu Regional Referral Hospital
Apiyo	Paska	M.D.	Gulu Regional Referral Hospital
Kiweewa	Francis	M.D., M.P.H.	Makerere University Lung Institute
Kirenga	Bruce	MBChB, NMED Ph.D.	Makerere University Lung Institute
Kimuli	Ivan	MBChB, NMED, M.P.H.	Makerere University Lung Institute
Atukunda	Angella	MBChB, NMED	Makerere University Lung Institute
Muttamba	Winters	MBChB, NMED	Makerere University Lung Institute
Remmy	Kyeyume	B.MLT.	Makerere University Lung Institute
Segawa	Ivan	B.Pharm.	Makerere University Lung Institute
Pheona	Nsubuga	B.Pharm., M.P.H.	Makerere University Lung Institute
Kigere	David	D.N.	Makerere University Lung Institute
Mbabazi	Queen Lailah	MBChB	Makerere University Lung Institute
Boersalino	Ledra	MBChB	Makerere University Lung Institute
Nyakoolo	Grace	B.Sc.	Makerere University Lung Institute
Kiweewa	Francis	MD., M.P.H.	Lira Regional Referral Hospital
Fred	Aniongo	B.MLT.	Lira Regional Referral Hospital
Alupo	Alice	R.N.	Lira Regional Referral Hospital
Ebong	Doryn	B.S.N.	Lira Regional Referral Hospital
Monday	Edson	B.S.N.	Lira Regional Referral Hospital
Nalubwama	Ritah Norah	M.D.	Lira Regional Referral Hospital
Kainja	Milton	M.D.	Lira Regional Referral Hospital
Ambrose	Munu	D.M.L.T.	Lira Regional Referral Hospital
Kwehayo	Vanon	R.N.	Lira Regional Referral Hospital
Nalubega	Mary Grace	R.N.	Lira Regional Referral Hospital
Ongoli	Augustine	MBChB	Lira Regional Referral Hospital

	Obbo	Stephen	MBChB, NMED, M.P.H.	Lira Regional Referral Hospital
	Sebudde	Nicholus	MBChB	Lira Regional Referral Hospital
	Alaba	Jeniffer		Lira Regional Referral Hospital
	Magombe	Geoffrey	B.Pharm.	Lira Regional Referral Hospital
	Tino	Harriet	B.Pharm., M.Sc.,P.H.S.M.	Lira Regional Referral Hospital
	Obonya, E.E.	Emmanuel		Lira Regional Referral Hospital
	Lutaakome	Joseph	M.D.	Masaka Regional Referral Hospital
	Kitonsa	Jonathan	M.D.	Masaka Regional Referral Hospital
	Onyango	Martin	M.D.	Masaka Regional Referral Hospital
	Naboth	Tukamwesiga	M.D.	Masaka Regional Referral Hospital
	Naluyinda	Hadijah		Masaka Regional Referral Hospital
	Nanyunja	Regina		Masaka Regional Referral Hospital
	Irene	Muttiibwa		Masaka Regional Referral Hospital
	Jane	Biira		Masaka Regional Referral Hospital
	Wimfred	Kyobejja		Masaka Regional Referral Hospital
	Leonard	Ssemazzi		Masaka Regional Referral Hospital
	Deus	Tkiinomuhisha		Masaka Regional Referral Hospital
	Babra	Namasaba		Masaka Regional Referral Hospital
	Taire	Paul		Masaka Regional Referral Hospital
	Lutaakome	Joseph	M.D.	St. Francis Hospital, Nsambya
	Nabankema	Evelyn	M.D.	St. Francis Hospital, Nsambya
	Ogavu	Joseph	M.D.	St. Francis Hospital, Nsambya
	Mugerwa	Oscar	M.D.	St. Francis Hospital, Nsambya
	Okoth	Ivan	M.D.	St. Francis Hospital, Nsambya
	Mwebaze	Raymond	M.D.	St. Francis Hospital, Nsambya
	Mugabi	Timothy	M.D.	St. Francis Hospital, Nsambya
	Makhoba	Anthony	M.D.	St. Francis Hospital, Nsambya
	Arikiriza	Phiona		St. Francis Hospital, Nsambya
	Theresa	Nabuuma		St. Francis Hospital, Nsambya
	Nakayima	Hope		St. Francis Hospital, Nsambya
	Frank	Kisuule		St. Francis Hospital, Nsambya
	Ramgi	Patrícia	M.D.	CISPOC: Centro de Investigação e Treino em Saúde da Polana Caniço, Maputo, Mozambique
	Pereira	Kássia	M.D., and all site team	CISPOC: Centro de Investigação e Treino em Saúde da Polana Caniço, Maputo, Mozambique
**companies**	Osinusi	Anu	M.D., M.P.H.	Gilead Sciences, Foster City, CA, USA
	Cao	Huyen	M.D.	Gilead Sciences, Foster City, CA, USA
	Klekotka	Paul	M.D., Ph.D.	Eli Lilly and Company, Indianapolis, IN
	Price	Karen	Ph.D.	Eli Lilly and Company, Indianapolis, IN
	Nirula	Ajay	M.D., Ph.D.	Eli Lilly and Company, Indianapolis, IN
	Osei	Suzette	M.D., Ph.D.	Vir Biotechnology / GlaxoSmithKline
	Tipple	Craig	M.B.B.S., M.R.C.P., Ph.D.	Vir Biotechnology / GlaxoSmithKline
	Wills	Angela	R.N., M.S.N., M.B.A.	Vir Biotechnology / GlaxoSmithKline
	Peppercorn	Amanda	M.D.	Vir Biotechnology / GlaxoSmithKline
	Watson	Helen	B.Sc., M.Sc.	Vir Biotechnology / GlaxoSmithKline

	Gupta	Rajesh	M.D.,M.S., M.P.H.	Vir Biotechnology / GlaxoSmithKline
	Alexander	Elizabeth	M.D., M.Sc., F.I.D.S.A.	Vir Biotechnology / GlaxoSmithKline
	Mogalian	Erik	Pharm.D., Ph.D.	Vir Biotechnology / GlaxoSmithKline
	Lin	Leo	M.D.	Vir Biotechnology / GlaxoSmithKline
	Ding	Xiao	Ph.D.	Vir Biotechnology / GlaxoSmithKline
	Margolis	David	M.D., M.P.H.	Brii Biosciences
	Yan	Li	M.D., Ph.D.	Brii Biosciences
	Girardet	Jean-Luc	Ph.D.	Brii Biosciences
	Ma	Ji	Ph.D.	Brii Biosciences
	Hong	Zhi	Ph.D.	Brii Biosciences
	Zhu	Quing	Ph.D.	Brii Biosciences
	Seegobin	Seth	PhD	AstraZeneca
	Gibbs	Michael	PhD	AstraZeneca
	Latchman	Mickel	BSc	AstraZeneca
	Hasior	Katarzyna	MSc	AstraZeneca
	Bouquet	Jerome	PhD	AstraZeneca
	Wei	Jianxin	PhD	AstraZeneca
	Streicher	Katie	PhD	AstraZeneca
	Schmelzer	Albert	PhD	AstraZeneca
	Brooks	Dennis	MD, PhD	AstraZeneca
	Butcher	Jonny	BSc	AstraZeneca
	Tonev	Dimitar	MD	AstraZeneca
	Arbetter	Douglas	MPH	AstraZeneca
	Damstetter	Philippe	MSc	AstraZeneca
	Legenne	Philippe	M.D	Molecular Partners and Novartis
	Stumpp	Michael	PhD	Molecular Partners and Novartis
	Goncalves	Susana	Pharm. D	Molecular Partners and Novartis
	Ramanathan	Krishnan	Ph.D	Molecular Partners and Novartis
	Chandra	Richa	M.D.	Molecular Partners and Novartis
**Leidos**	Baseler	Beth	M.S.	Leidos Biomedical Research, Inc., Frederick, MD, USA
	Teitelbaum	Marc	M.D.	Leidos Biomedical Research, Inc., Frederick, MD, USA
	Schechner	Adam	M.D.	Leidos Biomedical Research, Inc., Frederick, MD, USA
	Holley	H. Preston	M.D.	Leidos Biomedical Research, Inc., Frederick, MD, USA
	Jankelevich	Shirley	M.D.	Leidos Biomedical Research, Inc., Frederick, MD, USA
	Adams	Amy	M.S.	Leidos Biomedical Research, Inc., Frederick, MD, USA
	Becker	Nancy	B.S.N.	Leidos Biomedical Research, Inc., Frederick, MD, USA
	Dolney	Suzanne	B.S.N.	Leidos Biomedical Research, Inc., Frederick, MD, USA
	Hissey	Debbie		Leidos Biomedical Research, Inc., Frederick, MD, USA
	Simpson	Shelly	M.S.	Leidos Biomedical Research, Inc., Frederick, MD, USA
	Kim	Mi Ha	Ph.D.	Leidos Biomedical Research, Inc., Frederick, MD, USA
	Beeler	Joy	M.P.H.	Leidos Biomedical Research, Inc., Frederick, MD, USA
	Harmon	Liam	B.A.	Leidos Biomedical Research, Inc., Frederick, MD, USA
	Asomah	Mabel	M.S.H.S	Leidos Biomedical Research, Inc., Frederick, MD, USA
	Jato	Yvonne	M.P.H.	Leidos Biomedical Research, Inc., Frederick, MD, USA
	Stottlemyer	April	A.A.	Leidos Biomedical Research, Inc., Frederick, MD, USA
	Tang	Olivia	B.S.	Leidos Biomedical Research, Inc., Frederick, MD, USA
	Vanderpuye	Sharon	B.A.	Leidos Biomedical Research, Inc., Frederick, MD, USA
	Yeon	Lindsey	B.S.	Leidos Biomedical Research, Inc., Frederick, MD, USA
	Buehn	Molly	M.S.	Leidos Biomedical Research, Inc., Frederick, MD, USA
	Eccard-Koons	Vanessa	M.S.	Leidos Biomedical Research, Inc., Frederick, MD, USA

	Frary	Sadie	M.S.	Leidos Biomedical Research, Inc., Frederick, MD, USA
	MacDonald	Leah	M.S.	Leidos Biomedical Research, Inc., Frederick, MD, USA
	Cash	Jennifer	B.S.	Leidos Biomedical Research, Inc., Frederick, MD, USA
	Hoopengard ner	Lisa	M.S.	Leidos Biomedical Research, Inc., Frederick, MD, USA
	Linton	Jessica	M.S.	Leidos Biomedical Research, Inc., Frederick, MD, USA
	Schaffhauser	Marylu	B.A.	Leidos Biomedical Research, Inc., Frederick, MD, USA
	Nelson	Michaela	B.S.	Leidos Biomedical Research, Inc., Frederick, MD, USA
	Spinelli-Nadzam	Mary	B.S.	Leidos Biomedical Research, Inc., Frederick, MD, USA
	Proffitt	Calvin	M.A.	Leidos Biomedical Research, Inc., Frederick, MD, USA
	Lee	Christopher	B.S.	Leidos Biomedical Research, Inc., Frederick, MD, USA
	Engel	Theresa	M.F.S.	Leidos Biomedical Research, Inc., Frederick, MD, USA
	Fontaine	Laura	B.S.N.	Leidos Biomedical Research, Inc., Frederick, MD, USA
	Osborne	C.K.	B.S.	Leidos Biomedical Research, Inc., Frederick, MD, USA
	Hohn	Matt	M.B.A.	Leidos Biomedical Research, Inc., Frederick, MD, USA
	Galcik	Michael	M.S.	Leidos Biomedical Research, Inc., Frederick, MD, USA
	Thompson,	DeeDee	A.A.	Leidos Biomedical Research, Inc., Frederick, MD, USA
	Kopka	Stacey	M.S.	Leidos Biomedical Research, Inc., Frederick, MD, USA
	Shelley	Denise M.	M.S.	Leidos Biomedical Research, Inc., Frederick, MD, USA
	Mendez	Gregg	Ph.D.	Frederick National Laboratory for Cancer Research/Leidos Biomedical Research, Inc., Frederick, MD.
	Brown	Shawn	M.S.	Frederick National Laboratory for Cancer Research/Leidos Biomedical Research, Inc., Frederick, MD.
	Albert	Sara	M.P.H.	Leidos Biomedical Research, Inc., Frederick, MD, USA
	Balde	Abby	M.P.H.	Leidos Biomedical Research, Inc., Frederick, MD, USA
	Baracz	Michelle	M.S.	Leidos Biomedical Research, Inc., Frederick, MD, USA
	Bielica	Mona	M.Ed	Leidos Biomedical Research, Inc., Frederick, MD, USA
	Billouin-Frazier	Shere	M.Sc.	Leidos Biomedical Research, Inc., Frederick, MD, USA
	Choudary	Jay	M.B.A.	Leidos Biomedical Research, Inc., Frederick, MD, USA
	Dixon	Mary	A.A.	Leidos Biomedical Research, Inc., Frederick, MD, USA
	Eyler	Carolyn		Leidos Biomedical Research, Inc., Frederick, MD, USA
	Frye	Leanne	M.A.	Leidos Biomedical Research, Inc., Frederick, MD, USA
	Gertz	Jensen	M.B.A.	Leidos Biomedical Research, Inc., Frederick, MD, USA
	Giebeig	Lisa	M.S.	Leidos Biomedical Research, Inc., Frederick, MD, USA
	Gulati	Neelam	B.S.	Leidos Biomedical Research, Inc., Frederick, MD, USA
	Hankinson	Liz	B.S.	Leidos Biomedical Research, Inc., Frederick, MD, USA
	Hogarty	Debi		Leidos Biomedical Research, Inc., Frederick, MD, USA
	Huber	Lynda		Leidos Biomedical Research, Inc., Frederick, MD, USA
	Krauss	Gary	B.S.	Leidos Biomedical Research, Inc., Frederick, MD, USA
	Lake	Eileen		Leidos Biomedical Research, Inc., Frederick, MD, USA
	Manandhar	Meryan	M.P.H.	Leidos Biomedical Research, Inc., Frederick, MD, USA
	Rudzinski	Erin	B.S.	Leidos Biomedical Research, Inc., Frederick, MD, USA
	Sandrus	Jen	A.A.	Leidos Biomedical Research, Inc., Frederick, MD, USA
	Suders	Connie	M.B.A.	Leidos Biomedical Research, Inc., Frederick, MD, USA
**lab**	Natarajan	Ven	Ph.D.	Frederick National Laboratory for Cancer Research/Leidos Biomedical Research, Inc., Frederick, MD.
	Rupert	Adam W.	B.S., MT(ASCP)	Frederick National Laboratory for Cancer Research/Leidos Biomedical Research, Inc., Frederick, MD.
	Baseler	Michael	Ph.D.	Frederick National Laboratory for Cancer Research/Leidos Biomedical Research, Inc., Frederick, MD.
	Lynam	Danielle	M.S.	Frederick National Laboratory for Cancer Research/Leidos Biomedical Research, Inc., Frederick, MD.
	Imamichi	Tom	Ph.D.	Frederick National Laboratory for Cancer Research/Leidos Biomedical Research, Inc., Frederick, MD.

	Laverdure	Sylvain	Ph.D.	Frederick National Laboratory for Cancer Research/Leidos Biomedical Research, Inc., Frederick, MD.
	McCormack	Ashley	M.P.S.	Frederick National Laboratory for Cancer Research/Leidos Biomedical Research, Inc., Frederick, MD.
	Paudel	Sharada	Ph.D.	Frederick National Laboratory for Cancer Research/Leidos Biomedical Research, Inc., Frederick, MD.
	Cook	Kyndal	B.S.	Frederick National Laboratory for Cancer Research/Leidos Biomedical Research, Inc., Frederick, MD.
	Haupt	Kendra	B.S.	Frederick National Laboratory for Cancer Research/Leidos Biomedical Research, Inc., Frederick, MD.
	Khan	Ayub	Ph.D.	Frederick National Laboratory for Cancer Research/Leidos Biomedical Research, Inc., Frederick, MD.
	Hazen	Allison	M.S.	Frederick National Laboratory for Cancer Research/Leidos Biomedical Research, Inc., Frederick, MD.
	Badralmaa	Yunden	M.S.	Frederick National Laboratory for Cancer Research/Leidos Biomedical Research, Inc., Frederick, MD.
**ABML**	Smith	Kenneth		Advanced Biomedical Laboratories, LLC., Cinnaminson, NJ, USA
	Patel	Bhakti		Advanced Biomedical Laboratories, LLC., Cinnaminson, NJ, USA
	Kubernac	Amanda		Advanced Biomedical Laboratories, LLC., Cinnaminson, NJ, USA
	Kubernac	Robert		Advanced Biomedical Laboratories, LLC., Cinnaminson, NJ, USA
	Hoover	Marie L.	Ph.D.	Advanced Biomedical Laboratories, LLC., Cinnaminson, NJ, USA
	Solomon	Courtney		Advanced Biomedical Laboratories, LLC., Cinnaminson, NJ, USA
	Rashid	Marium		Advanced Biomedical Laboratories, LLC., Cinnaminson, NJ, USA
	Murphy	Joseph		Advanced Biomedical Laboratories, LLC., Cinnaminson, NJ, USA
**PCI**	Brown	Craig		PCI Pharma Services
	DuChateau	Nadine		PCI Pharma Services
	Ellis	Sadie		PCI Pharma Services
	Flosi	Adam		PCI Pharma Services
	Fox	Lisa		PCI Pharma Services
	Johnson	Les		PCI Pharma Services
	Nelson	Rich		PCI Pharma Services
	Stojanovic	Jelena		PCI Pharma Services
	Treagus	Amy		PCI Pharma Services
	Wenner	Christine		PCI Pharma Services
	Williams	Richard		PCI Pharma Services
